# Genome-Wide Sequence Characterization and Expression Analysis of Major Intrinsic Proteins in Soybean (*Glycine max* L.)

**DOI:** 10.1371/journal.pone.0056312

**Published:** 2013-02-20

**Authors:** Da Yong Zhang, Zulfiqar Ali, Chang Biao Wang, Ling Xu, Jin Xin Yi, Zhao Long Xu, Xiao Qing Liu, Xiao Lan He, Yi Hong Huang, Iqrar Ahmad Khan, Richard M. Trethowan, Hong Xiang Ma

**Affiliations:** 1 Institute of Biotechnology, Jiangsu Academy of Agricultural Sciences, Nanjing, China; 2 Department of Plant Breeding and Genetics, University of Agriculture, Faisalabad, Pakistan; 3 Cotton Research Institute, Shanxi Agricultural Sciences, Yuncheng, China; 4 Nanjing Agricultural University, Nanjing, China; 5 College of Agriculture & Applied Biology, University of Agriculture, Faisalabad, Pakistan; 6 Plant Breeding Institute, The University of Sydney, Sydney, Australia; Beijing Institute of Microbiology and Epidemiology, China

## Abstract

Water is essential for all living organisms. Aquaporin proteins are the major facilitator of water transport activity through cell membranes of plants including soybean. These proteins are diverse in plants and belong to a large major intrinsic (MIP) protein family. In higher plants, MIPs are classified into five subfamilies including plasma membrane intrinsic proteins (PIP), tonoplast intrinsic proteins (TIP), NOD26-like intrinsic proteins (NIP), small basic intrinsic proteins (SIP), and the recently discovered X intrinsic proteins (XIP). This paper reports genome wide assembly of soybean MIPs, their functional prediction and expression analysis. Using a bioinformatic homology search, 66 GmMIPs were identified in the soybean genome. Phylogenetic analysis of amino acid sequences of GmMIPs divided the large and highly similar multi-gene family into 5 subfamilies: GmPIPs, GmTIPs, GmNIPs, GmSIPs and GmXIPs. GmPIPs consisted of 22 genes and GmTIPs 23, which showed high sequence similarity within subfamilies. GmNIPs contained 13 and GmSIPs 6 members which were diverse. In addition, we also identified a two member GmXIP, a distinct 5^th^ subfamily. GmMIPs were further classified into twelve subgroups based on substrate selectivity filter analysis. Expression analyses were performed for a selected set of GmMIPs using semi-quantitative reverse transcription (semi-RT-qPCR) and qPCR. Our results suggested that many GmMIPs have high sequence similarity but diverse roles as evidenced by analysis of sequences and their expression. It can be speculated that GmMIPs contains true aquaporins, glyceroporins, aquaglyceroporins and mixed transport facilitators.

## Introduction

Water is essential for all living organisms. Like other living organisms, plant growth and development depends on water uptake and transport regulation across cellular membranes and tissues. For a long time, it was thought that water moved across cell membranes by free diffusion through a lipid bilayer. However, its transport was thought to be highly selective thus preventing uncontrolled movement of other solutes, protons, and ions. The first aquaporin gene (AQP1) was identified from human erythrocytes [1], and NOD26 from nitrogen-fixating symbiosomes in root nodules of soybean plants [2]. Since their discovery, many studies have indicated that aquaporins provide an important selective pathway for water transport across cellular membranes and they have changed our understanding of water flow regulation in plants under different physiological conditions [3], [4], [5].

Aquaporins are integral membrane proteins belonging to a large family of water channel proteins that assist the rapid movement of water across cellular membranes. Water and solute transport are universal requirements for living cells and these proteins are found in most organisms. Of the five AQP subfamilies, the original definition of the PIP and TIP subfamilies was based on their assumed location in the plasma membrane and tonoplast, respectively. When proteins of the TIP group were localized using antibodies, the signal was always confined to the tonoplast membrane fractions [6]. PIP localization seems less well defined. A PIP-family protein has been located to the plasma membrane in Arabidopsis [7]. PIPs to a small extent were detected in the plasma membrane of *M. crystallinum* but mostly in a vacuolar fraction in continuous sucrose gradients or, more likely, in a membrane fraction with a density similar to tonoplasts [8]. This is not surprising as it has been documented that cycling of mammalian AQPs between the plasma membrane and internal vesicles is under hormonal control [9]. The distribution of different TIPs in distinct plant vacuoles may also be based on similar mobility [10].

Aquaporins (AQPs) belong to the ancient major intrinsic proteins (MIPs) family found in animals, microbes, and plants. Since discovery of AQP1, 13 different AQPs have been identified in mammals while surprisingly a high number of their homologues have been found in plants such as 35 AQPs in *Arabidopsis* [11], [12], 31 full length expressed AQP genes in *Zea mays* [13], 33 in *Oryza sativa* [14], 23 in *Physcomitrella patens* [15], 37 in *Solanum lycopersicum* [16], and 71 in *Gossypium hirsutum* [17]. Plant AQPs sequence homologies are categorized into four subfamilies: the plasma membrane intrinsic proteins (PIPs), the tonoplast intrinsic proteins (TIPs), the nodulin26-like intrinsic proteins (NIPs) and the small and basic intrinsic proteins (SIPs) [13], [18]. However, in several dicots an uncategorized X intrinsic protein (XIPs), a novel AQP subfamily, has been reported [15]. For instance, on the basis of sequence homologies, 37 AQPs in tomato were classified into 18 PIP, nine TIP, six NIP, three SIP, and one novel XIP isoform [16].

Plant AQP gene expression is differentially regulated in various tissues and is also altered under different physiological and environmental stresses [11], [19]. AQP gene expression patterns in many plant species in specific tissues, cell types or in response to phytohormones or environmental factors has highlighted the putative role of water channels. AQPs play a central regulatory role in plant water relations and cellular water transport [20], [21]. AQPs mediate root water transport regulation in response to a variety of environmental stimuli and facilitate water transport from the roots through inner leaf tissues during transpiration and in expanding tissues [22]. AQPs also facilitate the transport of low molecular weight molecules like urea, boric acid, CO_2_ etc through the plant cell membrane [5], [23] and regulate assimilate transport in the phloem via sieve elements, stomatal control, movement of leaves, control of cytoplasmic homoeostasis etc [4], [5], [22]. Many different mechanisms appeared to be involved in regulation of plant aquaporin activity in cellular membranes. Beyond the initial regulatory alteration of gene expression based on plant cell type, developmental stage and environmental state, the subsequently translated aquaporins are sent to their target membrane, and then, when required, they facilitate the transmembrane flux of water and/or small non-electrolytes [24].

The first step in investigating the role of MIPs in soybean water relations is the identification of the MIP gene family. Therefore, the objective of this study is to identify soybean MIP genes and to investigate both their structural properties and expression patterns. In this study we identified 66 MIPs in the soybean genome. This paper presents their isoforms and genome-wide classification, and expression analysis specific to various tissues and water stress.

## Materials and Methods

### Identification of *GmMIPs*

A comprehensive search using the tblastn tool at www.phytozome.net/ across all the *Physcomitrella* and *Arabidopsis* MIPs was conducted. The CDS (Coding DNA Sequence) and putative protein sequences specific to soybean were downloaded using the BioMart online tool available at the website. Every sequence was individually compared with functional annotations by browsing the soybean genome database at www.phytozome.net/cgi-bin/gbrowse/soybean resulting in the identification of 66 MIPs for further analyses. The unclassified MIPs were classified into different isoforms by comparing the phylogenetic relationship of their putative protein sequences with clearly classified MIPs from soybean and *Arabidopsis* downloaded from http://www.phytozome.net/search.php?show=blast and http://www.uniprot.org/uniprot/, respectively.

### Multiple alignment, phylogenetic, and domain analysis

Sixty six MIPs were aligned together using ClustalX2 http://www.ebi.ac.uk/Tools/clustalx2/index.html. The untreated phylogenetic tree was constructed by the neighbor-joining method using TreeView software. The transmembrane regions were detected using the online tool available at http://www.ch.embnet.org/software/TMPRED_form.html.

### *In silico* subcellular predicted localization, gene expression analysis and computation of ka/ks value

The protein subcellular localization was predicted using the online tool WoLF PSORT available at http://wolfpsort.org/. The gene expression *in silico* was obtained by in putting the locus name using an on-line search tool at www.soybase.org. The Ka/ks values of the *GmMIPs* were calculated using the on-line computation service at http://services.cbu.uib.no/tools/kaks. Where, ka and ks are numbers of non-synonymous and synonymous substitutions per site, respectively. Ka/ks>1 indicates gene evolution under positive selection, Ka/ks<1 indicates purifying (stabilizing) selection and Ka/ks = 1 suggests a lack of selection or possibly a combination of positive and purifying selection at different points within the gene that cancel each other out.

### Identification of specificity determining positions (SDPs)

The aligned sequences of GmMIPs were inspected manually for SDPs following the prediction explained else where [25] and grouped into various function groups. The sequence of function groups were aligned using the ClustalW in GDE format. The SDPs were predicted using SDPpred (http://bioinf.fbb.msu.ru/SDPpred/algo.html; last accessed November 2012) and the positions where the Z-scores exceeded the Bernoulli estimator threshold were considered as SDPs [25].

### Plant materials and growing conditions

Seeds of soybean, *Glycine max* var. Sudou 3, were used to grow seedlings and extract total RNA for expression analysis of MIPs in the following experiments. The soybean plants were grown in 10 cm dia pots placed in the greenhouse/field at 28/25°C day/night temperatures, 12 h photoperiod and 75% humidity. The agronomic requirements of soybean were followed and kept uniform for all the plants.

### Expression analysis of GmMIPs in various tissues

The roots, stems, leaves, flowers and young pods were harvested separately from plants at the three leaf stage and total RNA extracted from the leaves, roots and stems and at maturity from flowers and pods. Semi-quantitative polymerase chain reaction (Semi-qPCR), as described in following paragraphs was used for expression analysis.

### Drought or no watering inducible expression analysis of GmMIPs

The soybean plants were grown following the protocols described in the preceding paragraphs. Water application was withheld at the three leaf stage. Roots were harvested at 0, 7, 14 and 21 d from both stressed (drought) and unstressed control plants for total RNA extraction. Semi-qPCR was used for expression analysis.

### Polyethylene glycol inducible expression analysis of GmMIPs

The soybean plants were grown following the protocols described above. At the three leaf stage, plants were carefully up-rooted to avoid root injury. Up-rooted plants were immediately transferred to 80 mL glass tubes containing 20% polyethylene glycol (PEG) and placed in the growth chamber. Up-rooted plants were also transferred to a control treatment in 80 mL glass tubes with no PEG. Roots were harvested at 0, 2, 4, and 12 h of PEG stress for RNA extraction. Real time or qPCR was used for expression analysis as per the conditions described in following paragraphs.

### Total RNA isolation and RT-PCR

The total RNAs were extracted from the collected samples for expression analyses using TRIzol® reagent (Invitrogen & Co.) following the manufacturer's instructions and quantified using Bio–Photometer (Eppendorf). The first-strand cDNA was synthesized through reverse transcription PCR (RT-PCR) using avian myeloblastosis virus (AMV) reverse transcriptase (Promega, USA). One µg RNA was used as a template to produce cDNA in a total reaction volume of 25 uL following the manufacturer's instructions.

### Semi-qPCR analysis

The reaction mixture of semi-quantitative polymerase chain reaction (semi-qPCR) consisted of 2.5 uL of 10× PCR buffer, 1.5 uL of 25 mmol/l MgCl_2_, 0.5 uL dNTP (10 mmol/1), 1.0 uL of each primer (10 µmol/1), 17.3 uL PCR-grade water, 0.2 uL(5 U/l) of rTaq, and 1.0 uL of the template consisted of reaction product (cDNA) from RT-PCR. The constitutive expression gene *GmTubulin* (accession number: XM_003550379, forward primer, 5′- AACCTCCTCCTCATCGTACT -3′; reverse primer, 5′- GACAGCATCAGCCATGTTCA -3′) was used as the internal control. The primer sequences, positions and expected product sizes are given in Table S1. The cycling parameters of semi-qPCR consisted of an initial denaturation at 94°C for 3 min; 27 subsequent cycles of denaturation at 94°C for 45 s, annealing at 55°C for 45 s, and extension at 72°C for 1 min; and finally extension at 72°C for 5 min. The qRT-PCR products were separated on a 1.0% agarose gel. The gel was viewed with a high performance CCD camera fixed in a Peiqing Gel photo system (Shanghai Peiqing Science and Technology Co., Ltd, Shanghai, China). The quantification of the bands and normalization was performed following [26]. Three independent repeats of the semi-qPCR experiments were carried out.

### Real time or qPCR

Real-time or qPCR was performed using a real-time PCR detection system (F. Hoffmann-La Roche Ltd, www.roche.com) with the SYBR® green supermix. Primers for qPCR were designed with the Primer Premier5.0 program (http://www.premierbiosoft.com/crm/jsp/com/pbi/crm/clientside/ProductList.jsp) and enlisted in Table S1. Sample preparation and qPCR analysis was conducted following SYBR® Premix Ex Taq™ (Perfect Real Time). A 10 µl of mix consisted of 5 µl of SYBR® Premix Ex Taq™ (TaKaRa Bio Inc; Shiga, Japan http://www.takara-bio.com/), 0.8 µl of each primer (forward and reverse, each 10 µmol/1), 1 µl of template consisted of reaction product (cDNA) from RT-PCR and 2.4 µl of d_2_H_2_O. The soybean GmPEPC gene (accession number: NM_001250673, forward primer, 5′-TTCCTTTATCAGAAATAACGAGTTTAGCT-3′; reverse primer, 5′-TGTCTCATTTTGCGGCAGC-3′) was used as an internal control or reference to detect the expression of the target MIPs [27]. An equal amount of cDNA template was used for each sample including the internal control. PCR amplification conditions were as follows: an initial denaturation step for 10 min at 95°C; 40 cycles of quantification consisting of denaturation for 10 s at 95°C, annealing for 20 s at 58°C, and extension for 30 s at 72°C; and completed by melting curve analysis to confirm the specificity of the PCR product. According to the manufacturer's instructions, similar results were obtained from relative gene expression data using the change in threshold cycle (ΔCt) (i.e., ΔCT) method described by Winer [28]. Specific gene expression levels were considered unavailable (N/A) if Ct (gene) >30 or <15. The qPCR analysis was repeated in three independent experiments.

## Results

### The soybean *GmMIP* genes, nomenclature and their distribution

**Figure 1:**
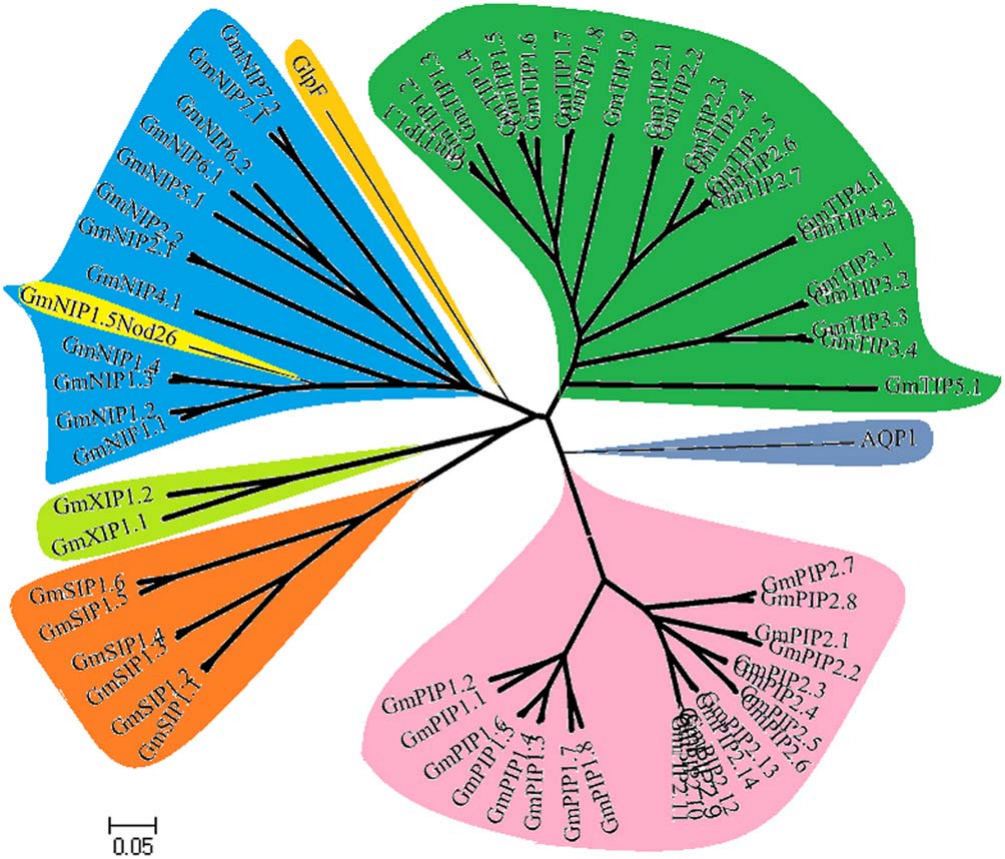
Phylogenetic analysis of 66 soybean (*Glycine max*) aquaporin proteins.

By mining the database of soybean *MIPs*, we identified 66 different GmMIPs (Table 1). The nomenclature of GmMIPs was established using phylogenetic relationships with known genes of *Physcomitrella patens*, *Arabidopsis thaliana*, *Zea mays* and *Oryza sativa* (Fig. S1) done previously for MIPs of other species. When aligned and compared by the Clustal-X/TreeView programs, the deduced protein sequences separated into five major branches (PIP, TIP, NIP, SIP and XIP; Fig. 1 and Fig. S2). These branches are consistent with current nomenclature developing in this field.

**Table 1:** Gene nomenclature to the identified soybean major intrinsic proteins (GmMIPs) loci and their locus name, location in genome, expressed sequence tag (EST), number of trans-membranes (NTM), polypeptide length (PL), *in silico* root specific expression (RSE), protein subcellular predicted localization (PSL) and ka/ks ratio.

Gene name	Locus name	Genome location	EST	NTM	PL (aa)	RSE	PSL	Ka/ks value	Comments
GmPIP1;1	Glyma03g14150	18018230..18021565	Yes	6	285	15	PLAS	<1	
GmPIP1;2	Glyma18g42630	51879812..51881980	Yes	6	305	23	PLAS	?	
GmPIP1;3	Glyma01g42950	54066066..54068057	Yes	6	287	51	PLAS	<1	
GmPIP1;4	Glyma11g02530	1656129..1658174	Yes	6	287	63	PLAS	<1	
GmPIP1;5	Glyma05g37730	41267148..41268807	Yes	6	288	296	PLAS	<1	
GmPIP1;6	Glyma08g01860	1202356..1204135	Yes	6	290	82	PLAS	<1	
GmPIP1;7	Glyma14g06680	4894197..4896207	Yes	6	290	1635	PLAS	<1	
GmPIP1;8	Glyma11g35030	36767510..36769078	Yes	6	290	1169	PLAS	<1	
GmPIP2;1	Glyma04g00450	227991..229365	Yes	6	276	96	PLAS	<1	
GmPIP2;2	Glyma06g00550	264336..265850	Yes	6	279	16	PLAS	<1	
GmPIP2;3	Glyma11g20600	17405538..17407774	Yes	6	287	300	PLAS	<1	
GmPIP2;4	Glyma12g08040	5736134..5738571	Yes	6	287	531	PLAS	<1	
GmPIP2;5	Glyma12g29510	32929324..32931027	Yes	6	288	324	PLAS	<1	
GmPIP2;6	Glyma13g40100	40664607..40666361	Yes	6	288	1008	PLAS	<1	
GmPIP2;7	Glyma03g33800	41279731..41281496	Yes	6	287	1	PLAS	<1	
GmPIP2;8	Glyma19g36530	43803906..43806142	Yes	6	286	1	PLAS	<1	
GmPIP2;9	Glyma02g08110	6345032..6348612	Yes	6	286	5	PLAS	<1	
GmPIP2;10	Glyma16g27130	31144909..31148555	Yes	6	286	39	PLAS	<1	
GmPIP2;11	Glyma16g27140	31154978..31156159	Yes	6	286	62	PLAS	<1	
GmPIP2;12	Glyma02g08120	6358474..6361413	Yes	6	286	1	PLAS	<1	
GmPIP2;13	Glyma10g35520	43763682..43766888	Yes	6	297	159	PLAS	<1	
GmPIP2;14	Glyma20g32000	40621201..40624089	Yes	6	285	106	PLAS	<1	
PseudoPIP#1	Glyma01g27970	37458238..37460664	?	5	254	0		<1	Not full length
PseudoPIP#2	Glyma02g42220	47295297..47297674	Yes	5	317	1082		1	2^nd^ NPA missing, Not full length
PseudoPIP#3	Glyma14g24430	29321210..29322268	?	4	188	0		>1	NPA modified
PseudoPIP#4	Glyma18g03330	2228741..2229353	Yes	3	128	2		?	1^st^ NPA missing, Not full length
GmTIP1;1	Glyma02g10520	8409966..8411440	Yes	6	253	0	CYTO	<1	
GmTIP1;2	Glyma18g52360	60989768..60991401	?	6	253	0	CYTO	<1	
GmTIP1;3	Glyma10g43680	50271428..50272965	?	7	253	0	PLAS	<1	
GmTIP1;4	Glyma11g15200	10892421..10894109	Yes	6	253	17	PLAS	<1	
GmTIP1;5	Glyma12g07120	4870480..4871652	?	6	246	0	PLAS	<1	
GmTIP1;6	Glyma13g40820	41270585..41271998	Yes	7	253	24	VACU	<1	
GmTIP1;7	Glyma03g34310	41779243..41780564	Yes	6	251	2244	CYTO	<1	
GmTIP1;8	Glyma19g37000	44258426..44259853	Yes	6	251	1181	CYTO	<1	
GmTIP1;9	Glyma13g20940	24436182..24438466	Yes	6	251	21	ER	<1	
GmTIP2;1	Glyma01g41670	53110677..53113455	Yes	6	250	693	PLAS	<1	
GmTIP2;2	Glyma11g03690	2476012..2478825	Yes	6	250	903	PLAS	<1	
GmTIP2;3	Glyma07g02060	1435523..1437651	Yes	6	249	14	VACU	<1	
GmTIP2;4	Glyma08g21730	16535219..16537122	Yes	6	249	15	VACU	<1	
GmTIP2;5	Glyma13g43250	43018922..43020336	Yes	6	248	1	PLAS	<1	
GmTIP2;6	Glyma15g02090	1393557..1395809	Yes	6	248	0	CYTO	<1	
GmTIP2;7	Glyma19g04450	4625496..4626575	?	6	238	0	PLAS	<1	
GmTIP3;1	Glyma09g28930	35913523..35915582	Yes	6	256	0	CYTO	<1	
GmTIP3;2	Glyma16g33530	36421819..36424304	Yes	6	256	0	MITO	<1	
GmTIP3;3	Glyma10g31750	40238530..40240337	Yes	6	255	0	CYTO	<1	
GmTIP3;4	Glyma20g35860	44068541..44070258	?	6	255	0	CYTO	<1	
GmTIP4;1	Glyma04g08830	6943153..6944783	Yes	6	247	357	VACU	<1	
GmTIP4;2	Glyma06g08910	6498818..6500103	Yes	6	247	89	CYTO	<1	
GmTIP5;1	Glyma09g35860	41742635..41743884	?	6	248	0	CHLO	<1	
PseudoTIP#1	Glyma15g04630	3223182..3223750	?	5	153	0			2^nd^ NPA missing
PseudoTIP#2	Glyma10g06750	5471639..5473323	?	5	189	0			2^nd^ NPA missing
PseudoTIP#3	Glyma12g01490	895186..897034	?	5	188	0		<1	Not full length
GmNIP1;1	Glyma05g29510	35105884..35108185	Yes	6	271	2	VACU		
GmNIP1;2	Glyma08g12660	9268559..9270946	Yes	6	275	1	PLAS		
GmNIP1;3	Glyma13g29690	32551102..32553703	Yes	6	274	0	PLAS	<1	
GmNIP1;4	Glyma15g09370	6704209..6706791	Yes	6	268	0	PLAS	<1	
GmNIP1;5Nod26	Glyma08g12650	9262302..9265834	Yes	6	272	0	PLAS		
GmNIP2;1	Glyma09g37280	42824943..42829709	Yes	6	294	279	PLAS		
GmNIP2;2	Glyma18g49410	58816436..58821548	Yes	6	296	124	EXTR		
GmNIP4;1	Glyma07g34150	39062920..39065820	?	6	269	0	VACU	<1	
GmNIP5;1	Glyma10g36560	44670892..44676555	Yes	6	291	38	PLAS		
GmNIP6;1	Glyma08g23230	17701761..17706495	Yes	6	307	1	PLAS	<1	
GmNIP6;2	Glyma15g00620	355676..359967	Yes	6	305	15	PLAS	<1	
GmNIP7;1	Glyma02g15870	14348789..14351092	?	6	294	0	PLAS	?	
GmNIP7;2	Glyma10g03870	2898450..2900795	?	6	277	0	PLAS		
PseudoNIP#1	Glyma02g41400	46541265..46543675	?	7	216	0		<1	Not full length
PseudoNIP#2	Glyma05g29500	35100591..35104790	Yes	5	244	0			TM5 missing, but rest similar to GmNIP1;1
PseudoNIP#3	Glyma07g02760	1873672..1881683	?	5	182	0			Not full length
PseudoNIP#4	Glyma07g02800	1909545..1912431	Yes	4	185	0			Not full length
PseudoNIP#5	Glyma07g03030	2087864..2090380	?	4	249	0			Not full length
PseudoNIP#6	Glyma13g01800	1483837..1490592	?	5	227	0			1^st^ NPA missing, Not full length
PseudoNIP#7	Glyma14g07560	5711153..5714115	?	7	217	0		<1	Not full length
PseudoNIP#8	Glyma14g35030	43721841..43723560	?	7	220	0			Not full length
PseudoNIP#9	Glyma20g01750	1269856..1275738	?	6	239	0			1^st^ NPA missing, Not full length
PseudoNIP#10	Glyma20g31040	39698455..39705450	?	5	264	42			Not full length
PseudoNIP#11	Glyma08g29500	23981039..23981867	?	2	92	0			2^nd^ NPA missing, Not full length
PseudoNIP#12	Glyma14g13260	12544663..12544842	?	2	60	0			1^st^ NPA missing
GmSIP1;1	Glyma02g07680	6061309..6065568	Yes	6	248	1	VACU	<1	
GmSIP1;2	Glyma16g26720	30813218..30817735	?	6	246	1	VACU	<1	
GmSIP1;3	Glyma19g28430	35912781..35923174	Yes	6	249	27	PLAS	<1	
GmSIP1;4	Glyma16g04800	4096288..4102424	Yes	6	249	23	PLAS		
GmSIP1;5	Glyma12g10430	8369034..8369846	Yes	6	240	0	PLAS	<1	
GmSIP1;6	Glyma06g46340	48987251..48988278	Yes	6	240	1	PLAS		
PseudoSIP#1	Glyma01g04520	4099619..4100401	?	2	141	0		>1	2^nd^ NPA missing, Not full length
PseudoSIP#2	Glyma03g27340	35075328..35078322	Yes	5	231	5		<1	Not full length
PseudoSIP#3	Glyma19g30320	37949307..37951724	Yes	5	237	10		<1	Not full length
GmXIP1;1	Glyma11g10360	7439903..7440754	?	6	271	0	EXTR	<1	
GmXIP1;2	Glyma12g02640	1729006..1730580	Yes	6	313	0	CYTO		
PseudoXIP#1	Glyma11g10350	7436965..7438428	?	5	202	23			Not full length

Where, ka and ks are numbers of nonsynonymous and synonymous substitutions per site, respectively. PLAS: plasma membrane. VACU: vacuolar membrane, CYTO: cytosol, ER: endoplasmic reticulum, MITO: mitochondrion, CHLO: chloroplast and EXTRA: extra-cellular.

A comprehensive phylogenetic analysis was conducted to establish groups of homology within the GmMIP gene family. The 66 GmMIPs were classified into various subfamilies including 22 GmPIPs, 23 GmTIPs, 13 GmNIPs, six GmSIPs and two new GmXIPs i.e., uncharacterized isoforms (Fig. 1 and Tables 1 & 2). The GmXIPs might be similar to the novel plant AQP subfamily XIP recently reported in moss [15] and tomato [16]. All the members of the GmPIPs subfamily localized to plasma membranes (Table 1). The predicted localization of members of the GmTIPs subfamily was diverse, and predicted localization included cytosol, plasma membrane, endoplasmic reticulum, vacuole, mitochondria and chloroplast. From the GmNIPs subfamily, GmNIP1;1 and GmNIP4;1 localized to vacuoles, GmNIP2;2 to extra-cellular structures and the rest to plasma membranes. Two members of the GmSIP subfamily localized to vacuoles and the remainder to plasma membranes. GmXIP1;1 localized to extra-cellular structures and GmXIP1;2 to cytosol. The ka/ks ratio was >1 and 1 for PseudoPIP#3 and PseudoSIP#1, and PseudoPIP#2, respectively. The remaining GmMIPs showed ka/ks ratio <1 (Table 1, also see Table S2 and Fig. S3 for details).

**Table 2:** Distribution of GmMIPs on soybean chromosomes.

Chromosome	PIPs	TIPs	NIPs	SIPs	XIPs	Total
1	1	1				2
2	2	1	1	1		5
3	2	1				3
4	1	1				2
5	1		1			2
6	1	1		1		3
7		1	1			2
8	1	1	3			5
9		2	1			3
10	1	2	2			5
11	3	2			1	6
12	2	1		1	1	5
13	1	3	1			5
14	1					1
15		1	2			3
16	2	1		2		5
17						0
18	1	1	1			3
19	1	2		1		4
20	1	1				2
Total	22	23	13	6	2	66

The GmMIPs were distributed throughout the soybean genome except chromosome 17 (Table 2). All chromosomes carried at least one (chromosome 14) and a maximum of six (chromosome 11) GmMIPs. Thirty one GmMIPs are on the + strand of double stranded DNA.

### Exon-intron structure analysis

**Figure 2:**
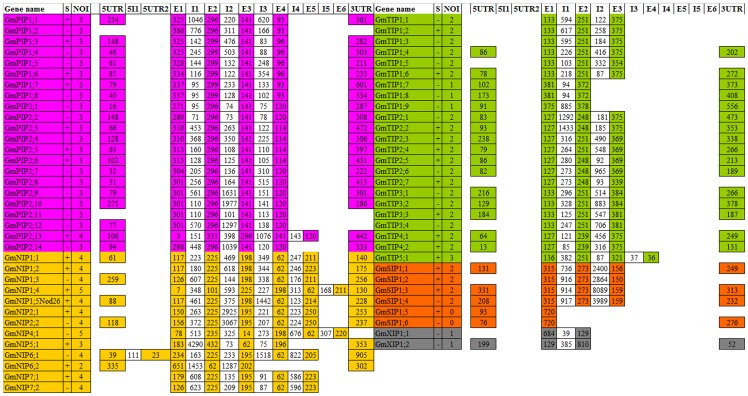
Exon-intron structure analysis of 66 soybean aquaporins. S stands for strand, NOI for number of introns, E for exon and I for intron.

The 66 GmMIP sequences were also analyzed for distribution of introns and exons; the results are shown in Fig. 2 (also see Fig. S4). The number of introns ranged from zero (in GmSIP1;5 and GmSIP1;6) to 5 (in GmNIP1;4 and GmNIP4;1). The division into five subfamilies based on comparison of the deduced protein sequences (see above) was mirrored in the intron-exon structures. All GmPIPs included three introns except GmPIP2;13 which contained four introns (intron # 1 is additional intron). For the GmTIPs, two introns were usual, but three genes: GmTIP1;7, GmTIP1;8 and GmTIP1;9 contained only one intron while one gene GmTIP5;1 contained three introns. GmNIPs contained variable introns with the majority characterized by 4 introns. GmNIP5;1 lacked one intron (intron # 2), GmNIP6;2 lacked two introns (intron # 2 and 3) while GmNIP1;4 and GmNIP4;1 each had five introns. GmSIPs contained two introns except GmSIP1;5 and GmSIP1;6 which included no intron. GmXIPs have a single intron. All the AtPIPs, PtPIPs and some of OsPIPs have three introns, however, some OsPIPs contained fewer and one member OsPIP2;8 contained no intron [29], [30]. In AtTIPs, PtTIPs and OsTIPs, the pattern was more varied; the majority of the genes contained two introns and the others either one or none. Most of AtNIPs, PtNIPs and OsNIPs were observed to have four introns while the AtSIPs, PtSIPs and OsSIPs have two [29], [30].

The intron insertion positions were different among the five sub-families and also varied within sub-families. Intron length varied widely in the range of 30 to 8089 nucleotides. The length of each exon was similar for most members in each subfamily, however, deviations were also noted.

### Paradigm of GmMIPs function

The MIPs specificity as a true water facilitator (AQP) or a glycerol facilitator (GlpF) or transporter of other elements such as ammonia, boron, urea etc, was characterized following previously deduced rules of sequence comparison [31], aquaporin specificity and phosphorylation sites [32], aromatic/argenine (ar/R) [33] and non-aqua substrates specificity [25], [34] filters. Multiple alignments were carefully inspected to identify residues that are directly linked to substrate specificity/function.

#### MIP's selectivities/specificities

**Figure 3:**
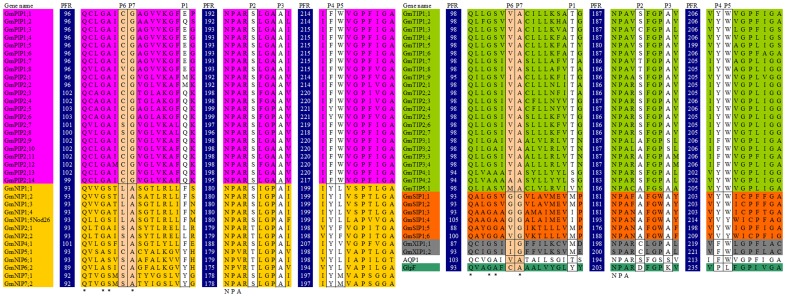
Portion of the 66 multiple sequence alignment. PFR stands for position of first residue in each sequence segment. The positions P1 to P5 predicted to have a functional role in MIP proteins are boxed. The positions P6 and P7 indicate the positions of residues to differentiate between the subfamilies.

Five discriminating positions were identified, which were conserved within each subfamily but differed between the subfamilies. These positions are located in highly conserved regions, and can be easily retrieved from any sequence (Fig. 3 and Table 3). Table 3 showed that five discriminating residues and two highly conserved NPA domains characterized all GmMIPs as water facilitators with some controversy in GmNIPs. Some GmNIPs have residues which are characteristics of GlpF-type.

**Table 3:** Consensus residues distinguishing AQPs and GlpF proteins.

Consensus position*	P1 at TP of H3	P2 2^nd^ residue after 2^nd^ NPA	P3 6^th^ residue after 2^nd^ NPA	P4 at last H	P5 at last H	P6 at H3	P7 at H3	1^st^ NPA	2^nd^ NPA
AQP-type	T/A/M/Q/E/V	S/A	A/S	F/Y	W/I/L	V	A	NPA	NPA
GlpF-type	Y/F	D	R/K	P/A	I/V/A/L/M/W	C	A	NPA	NPA
*Glycine max* MIP family									
GmPIP	Q/E/M	S	A	F/Y	W	C/S/V	G	NPA	NPA
GmTIP	T/S/V	S/A/T/C	A/S	Y/F	W	A/I/M/T/V	A	NPA	NPA
GmNIP	F/Y/L	S/T	A	Y	L/F/M/I/V	C/L/S	A	NPA/S	NPA/V
GmSIP	M/I	A	A	Y/V	W/Y	G/V	G	NPT/S	NPA
GmXIP	M	C	A	F	W	I	G	N/SPV/I	S/NPA
Character									
AQP-like	PIP, TIP, SIP, XIP	PIP, TIP, NIP, SIP	PIP, TIP, NIP, SIP, XIP	PIP, TIP, NIP, SIP, XIP	PIP, TIP, SIP, XIP	PIP, TIP, SIP, XIP			
GlpF-like	NIP	-	-	-	NIP	PIP, NIP			

H, transmembrane domain. P1–P7, conserved residues that may distinguish functions.

We also identified two adjacent additional residues within the same highly conserved region on the basis of 66 GmMIP sequences which in combination characterize the five subfamilies. In H3 transmembrane, the 6^th^ and 7^th^ residues following the most conserved Q residue of AQP1 and GlpF are VA and CA respectively. These residues characterized GmPIPs with C/S/V and G, GmTIPs with A/I/M/T/V and A, GmNIPs with C/L/S, and A, GmSIPs with G/V and G, and GmXIPs with I and G, respectively.

**Figure 4:**
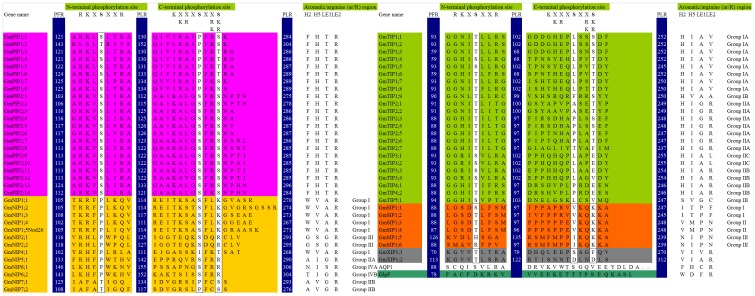
Phosphorylation sites in C-terminal domain, N-terminus and aromatic/arginine (ar/R) region in *GmMIPs*.

AQP1 and GlpF were used as structural templates the comparison of homology of the ar/R region in GmMIPs. In AQP1, the ar/R region is formed by Phe-58 (H2), His-182 (H5), Cys-191 (LE1), and Arg-197 (LE2; Fig. 4). In all the GmPIPs, the ar/R region is formed by Phe (H2), His (H5), Thr (LE1), and Arg (LE2). An examination of the ar/R region of GmPIPs shows close similarity to AQP1, however, the LE1 residue is Thr rather than Cys.

GmTIPs show three different ar/R subgroups: GmTIP Group IA (GmTIP1;1–8), IB (GmTIP1;9), GmTIP Groups IIA (GmTIP2;1–7), IIB (GmTIP3;3–4, and GmTIP4;1–2) and IIC (GmTIP3;1–2), and GmTIP Group III (GmTIP5;1). Homology comparisons of GmTIPs from Groups I and II show that the ar/R regions have a conserved His residue at the H2 position and a conserved Ile residue at the H5 position. His at the H2 position is replaced by Ser in Group III while Group IB and III possess Val at the H5 position. The loop E residues of GmTIPs at LE1 position is either an Ala (Group I, IIB and IIC) or a Gly (Group IIA and III). At LE2, Group I GmTIPs contain Val, Groups IIA and IIB contain the highly conserved Arg residue, Group IIC contains Leu and Group III contains Cys.

Nodulin 26 is a well-studied GmNIP, which has been identified as aquaglyceroporin. Six GmNIPs (GmNIP1;1–5 and GmNIP4;1, Group I) possess a conserved ar/R tetrad motif of the Nodulin 26. Group IIA (GmNIP5;1) and IIB GmNIPs (NIP7;1–2) have a divergent ar/R tetrad with the substitution of an Ala for Trp at position H2 and Gly for Ala at LE1. Group IIA has further substitution of Ile for Val at H5 position. Group III GmNIPs (GmNIP2;1–2) deviate from Group II with substitution of Gly and Ser at H2 and H5 positions. GmNIP6;1 is divergent from Group II with substitution of Asn and Ser at H2 and LE1 positions respectively, while in GmNIP6;2 Thr replaced Ala at H2 position. GmNIP5;1, GmNIP6;1 and GmNIP6;2 also possess a substitution within the NPA motif in loop E, a bulkier Val residue substituted the conserved Ala residue (i.e. NPV).

Analysis of the ar/R regions of GmSIPs suggests that three different combinations of residues are formed, Group I (GmSIP1;1–2), Group II (GmSIP1;3–4) and Group III (GmSIP1;5–6). Group I shows Ile, Thr, Pro and Phe residues at H2, H5, LE1 and LE2, respectively. The residues Val, Met, Pro and Asn in Group II are present at H2, H5, LE1 and LE2, respectively. In Group III, Asn and Ile replaced Val and Met of Group II at H2 and H5 positions. The GmSIP1;1–4 possess the NPT sequence and the GmSIP1;5–6 have the NPS sequence in place of the characteristic first NPA motif in loop B.

The GmXIPs show divergent ar/R region, three residues at H2, H5 and LE1 are different from AQP1 and GlpF. The residues at H2 and H5 are Val and Ile respectively in both the GmXIPs. At LE1 position, Ala replaced Val in GmXIP1;2 compared to GmXIP1;1. GmXIP1;2 also contains a substitution within both NPA motifs, Ile replaced Ala in first NPA while Ser replaced Asn in second NPA motif. GmXIP1;1 has substitution within first NPA motif where Ser and Val replaced respective residues of Asn and Ala (i.e., SPV).

All GmMIPs were analyzed for putative specificity determining positions (SDPs) for non-aqua substrate [25]. SDPs of five substrates (ammonia, boric acid, CO_2_, H_2_O_2_, and urea) were observed while SDPs for silicic acid were not observed in GmMIPs (Table 4). Most of GmPIPs, GmTIPs and GmNIPs contained these SDPs while none were present in GmSIPs and GmXIPs (Fig. S5).

**Table 4:** Putative specificity determining positions (SDPs) in GmMIPs for non-aqua substrates.

Subtrates		SDP1–4	SDP5	SDP6–9
Ammonia	Position	Loop C	Loop E	C
	Filter	Fxx(K/N)xxxFxxT	**A**	DxxLxExT
Boric acid	Position	TM2/Loop B	TM4	TM5
	Filter	Txx IxxxHxx**P**	E	LxxLxT/AxP
Co_2_	Position	TM3	Loop D	Loop E/TM6
	Filter	L/V/IxxIxxxCxxA	I	DxxWxDxW
H_2_O_2_	Position	TM3	TM5	Loop E/TM6
	Filter	A/SxxA/GxxxL/VxxA/F/L/V	I/V	H/I/L/QxxF/YxA/VxP
Urea	Position	Loop B	TM4	TM5/Loop E
	Filter	Hxx**P**xxxF/I/LxxA/F	L	A/PxxG/SxG/Sx**N**

TM, transmembrane.

#### Regulation of MIPs functions

The phosphorylation sites in C-terminal domain and N-terminus were detected and presented in Fig. 4. A highly conserved Ser residue is present in the N-terminal motif, RKXSXXR/K or only KXSXXR/K, which is conserved in all GmPIPs except GmPIP1;2. This motif is also conserved in GmTIPs, GmNIPs, GmSIPs and GmXIPs but usually Ser is replaced by Thr in GmTIPs, GmSIPs and GmXIPs, and by Pro in GmNIPs. Thr also replaced Ser in GmPIP1;2. His and Arg replaced Ser in GmSIP1;5 and GmSIP1;6 respectively. The C-terminal motif SFRS is present in all GmPIP2 subfamily members while the PFK/ST/S motif is present in all GmPIP1 subfamily members. The motif, KXXSXXK, is present in GmNIP1 subfamily members including NOD26, the KSXXR motif in GmNIP2 subfamily members, KIFKT in GmNIP4;1, the motif XSFRR in GmNIP5;1, GmNIP6;1 and GmNIP6;2, and XPFCS in GmNIP7 subfamily members. All GmTIPs, GmSIPs and GmXIPs subfamily members lack this phosphorylation motif in the C-terminal region.

### Expression of *GmMIP* genes in various plant organs

**Figure 5:**
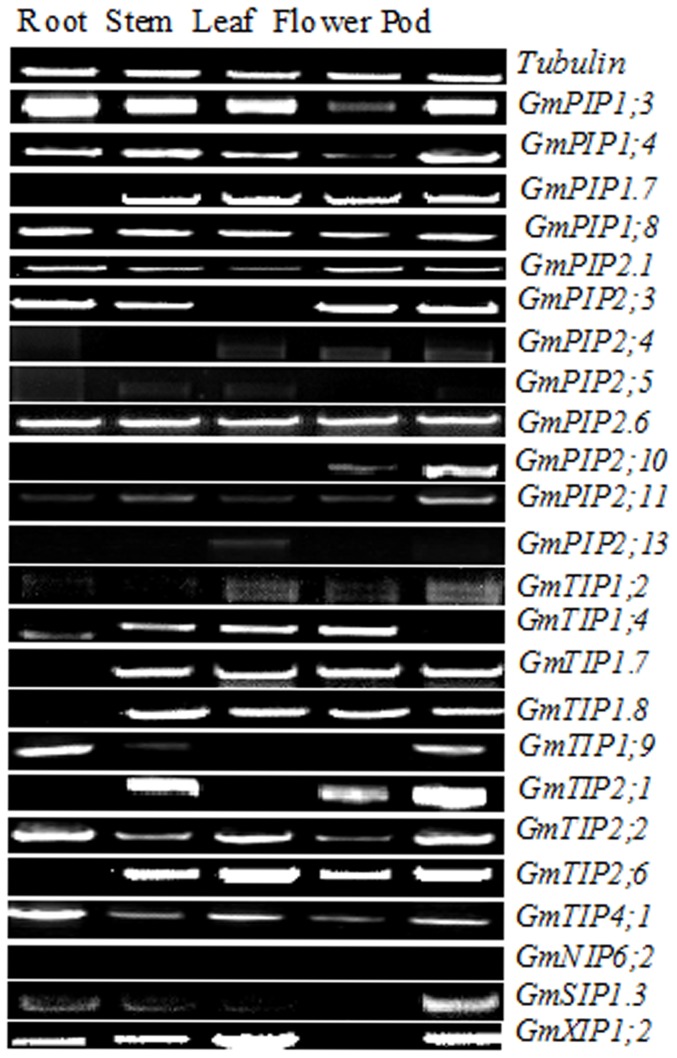
Expression patterns of 25 *GmMIPs* in various organs root, stem, leaf, flower and pod. These *GmMIPs* were selected for tissue specific expression analysis by semi-qPCR on the basis of *in silico* root specific expression (>50, see Table 1) and phylogenetic relationship (see Fig. 1).

The *in silico* expression of 23 GmMIPs in roots was >50 (Table 1). Based on *in silico* root specific expression and phylogenetic relationship (Fig. 1), 24 *GmMIPs* were selected for tissue specific expression analysis by semi-qPCR (Fig. 5). The expression patterns of GmMIPs in various organs of soybean were identified. GmPIP1;3 strongly expressed in roots, stems, leaves and pods. Other strongly expressed genes included GmPIP1;4 in stems and pods, GmPIP2;3 in roots, flowers and pods, GmTIP1;4 in flowers, GmTIP1;7 in stems, leaves, flowers and pods, GmTIP1;9 in roots, GmTIP2;1 in stems and pods, GmTIP2;2 in roots and pods, GmTIP2;6 in stems, leaves, flowers and pods and GmXIP1;2 in leaves. GmPIP2;6 and GmTIP4;1expressed marginally while GmPIP2;11 was marginally lower in all plant parts. The rest of the GmMIPs gave weak, marginal, marginal low or null expression.

### Screening for dehydration-inducible *GmMIP* genes

**Figure 6:**
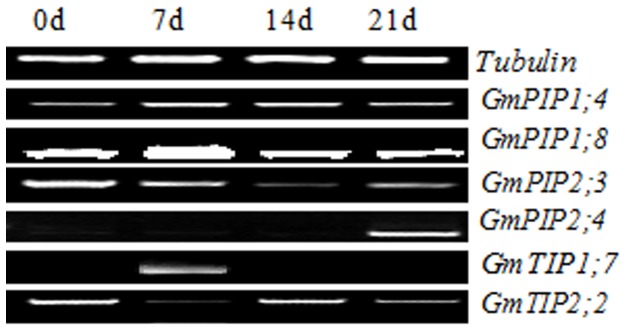
Expression patterns of six *GmMIPs* in roots of soybean at 0, 7, 14 and 21 days of no watering. The six MIPs were selected from 25 GmMIPs expressing in root (see Fig. 5) and distributed on chromosomes 11, 12 and 13 (see Table 1) as each of these chromosomes contain highest number of MIPs. The expression pattern of control plants (water was applied after three-leaf stage till 21 d) at various time points was similar to those at 0 d of no watering and thus picture not presented.

The expression patterns of selective *GmMIPs*, expressing in root (Fig. 5) and distributed on chromosomes 11, 12 and 13 (Table 1), were further analyzed at 0, 7, 14 and 21 d of water stress using semi-qPCR (Fig. 6). Tubulin was used as the internal control. As GmTIP1;7 is an isoform of AtTIP1;1 or AtTIP1;2 which are known as salt induced tonoplast intrinsic protein (SITIP), thus it was specifically included for semi-qPCR analysis under drought. Most of the *GmMIPs* expressed stably in roots with slight deviations at various times of water stress. The expression of GmPIP1;8 peaked significantly at 7 d of stress whereas GmTIP1;7 only expressed at 7 d without watering and GmPIP2;4 only after 21 d of water stress.

**Figure 7:**
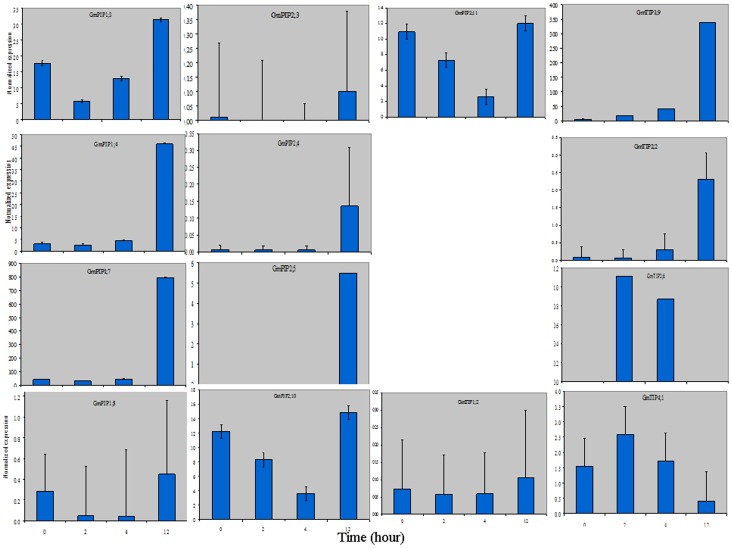
Normalized expression of GmMIPs at 0, 2, 4 and 12 h after 20% PEG treatment compared to internal control. The control plants showed non-significant deviations at various time points from those at 0 h and thus picture not presented.

We compared the expression levels of 14 GmMIP genes with GmPEPC (internal control) in roots of soybean by qPCR at 0, 2, 4 and 12 h of 20% PEG treatment (Fig. 7). The relative expression (RE) of GmPIP1;4, GmPIP1;7, GmPIP2;3, GmPIP2;4, GmPIP2;5, GmTIP1;9 and GmTIP2;2 significantly increased (>2-fold) at 12 h compared to 0 h PEG stress. However, GmPIP2;3 first showed a significant decrease in RE before peaking at 12 h PEG stress. GmPIP1;4, GmPIP1;7 and GmTIP1;9 showed a gradual increase from 0 to 4 h followed by a rapid increase at 12 h. GmPIP1;8 showed significant decrease in RE at 2 and 4 h PEG stress. The expression pattern of GmPIP1;3 decreased 2 h after application of PEG, and began to increase at 4 h and peaked at 12 h after application. The expression of GmPIP2;10 and GmPIP2;11 decreased gradually to 4 h and expression was maximized 12 h post application of PEG. The expression pattern of GmTIP4;1 increased at 2 h and decreased gradually with minimum expression at 12 h. GmTIP2;6 expressed 2 and 4 h after application of PEG stress. The RE of the remaining GmMIPs fluctuated non-significantly at various time points after application of PEG stress.

## Discussion

### Paradigm of GmMIPs function

The water channel protein family is part of the MIP superfamily of proteins. MIP proteins are highly diversified in plants and thus likely influence plants responses to external stresses. Most, if not all MIP proteins can be classified into two physiological groups: (1) AQPs to transport water and (2) GlpF to transport small neutral solutes such as glycerol [31]. There is a general view that most AQPs in plants regulate water flow and that a subset may facilitate the movement of glycerol or other small molecules [22], [35]. Considering these two main functions and following the rules of sequence comparison, aquaporins specificity and phosphorylation sites, and ar/R selectivity filters [31], [32], [33], [34], the present work focuses on the characterization of functional residues in the MIP proteins found in *Glycine max*. Most MIPs found in soybean are AQP type.

We identified a total of 66 aquaporin genes in soybean. The number of aquaporin genes described in this study is double that of rice, maize, tomato and Arabidopsis. It can be speculated that the palaeopolyploid nature of soybean resulted in duplication of these genes across the genome. These 66 genes separated into five subfamilies. The most frequent subfamilies observed were GmPIP and GmTIP, which contained 22 and 23 genes respectively (Fig. 1 and Table 1). The deduced rules of putative protein sequence comparison showed that all GmPIPs and GmTIPs have features similar to AQPs, however, GmPIPs residues at P6 (in H3) are similar to GlpF (Table 3, Fig. 3). The N-terminal phosphorylation site and ar/R selectivity filter showed that all GmPIPs are like AQP (Fig. 4). The C-terminus phosphorylation site in GmPIP1 subfamily members is not similar to AQP type where Pro replaced Ser. Members of GmPIP2 are similar to AQP (Fig. 4). AtPIP2 group are described as good AQPs in the *Xenopus laevis* expression system, whereas AtPIP1 proteins often cause lower osmotic water permeability (Pf) values in this expression system [36]. It is speculated that AtPIP1 AQPs could be responsible for the transport of yet unidentified solutes across the plasma membrane.

Twenty three GmTIPs were classified into three subgroups based on ar/R filter (Fig. 4). AtTIPs have been classified into three groups [33]. Subgroup IA and IIC are unique in the present studies. The NtTIP1 have been shown to transport water, glycerol, and urea when expressed in *X. laevis* oocytes [37].

All GmNIPs were classified into four subgroups. GmNIP2 and GmNIP6 subfamily members grouped in subgroup III and IV respectively appeared novel based on ar/R filter. NIPs have previously emerged as an interesting AQP subclass in terms of transport specificity and they are subdivided in two subgroups based on the predicted structure of their selectivity filter [33], [38]. It has been reported that phosphorylation of Nodulin-26 on Ser 262 enhanced water permeability and that phosphorylation is stimulated by drought [39]. AtNIPs have been reported to be classified in two subgroups [33]. Mixed transport activities of NIPs have been observed in different organisms, for example, GmNOD26 can form a functional water channel and produce glycerol permease in *X. laevis* oocytes [40]. The AtNIP1;1 was predicted as an AQP when expressed in *X. laevis* oocytes [41]. The amino acids for ar/R filter in subgroup III consisted of Gly, Ser, Gly, and Arg (GSGR), compared with Ala, Ile, Gly, and Arg (AIGR) in subgroup IIA (Fig. 4), which are comparable with OsLsi1 and AtNIP5;1 respectively. The residue at the H5 position of the ar/R filter of both OsLsi1 and AtNIP5;1 were revealed to play a key role in membrane permeability to silicic acid (Si) and boric acid (B), although there is a relatively low selectivity for arsenite (As) [34]. Previous reports show that G/A substitution at H2 (GSGR to ASGR) does not affect the transport activity of OsLsi1 for Si, As, B and water. The S/I substitution (GSGR to GIGR or AIGR) at H5 resulted in loss of Si, B and water transport activity. However, as transport was by 60% instead of total loss. AtNIP5;1, with AIGR residues for ar/R filter, was able to transport boric acid, water, and arsenite, but not silicic acid [34]. A single or double mutation at H2 and/or H5 did not result in any Si transport activity.. In contrast, both single and double mutations at the H2 and/or H5 positions showed As transport activity and H5 I/S substitution led to decreased B transport activity while water permeability remained unaffected. The GmNIPs in subgroup III can be speculated to transport Si, As, B and water while those in subgroup IIA may transport As, B and water.

The GmSIP subfamily contained six diverse members, which separated into three unique subgroups based on ar/R selectivity filter. The GmXIP subfamily contained two members which are also different. The members of the GmNIP and GmSIP subfamilies were divergent while those of the GmPIP subfamily were more similar (Fig. 1). Similarity among members of the PIP subfamily and divergence observed in the NIP subfamily has also been reported in Arapidopsis [12], tomato [16] and cotton [17]. The distribution of GmMIPs between the five subfamilies in soybean was similar to observations in species such as Arabidopsis, rice, maize etc. However, the two member subfamily of GmXIP has only recently been reported in moss [15] (see Fig. S1 for comparison of XIPs of soybean and moss), tomato [16] and cotton [17], and is present in mosses and dicots and is lost in monocots [15]. Some GmNIPs and a few GmPIPs have characteristics of both AQP and GlpF, and can be called as aquaglyceroporin [33], [42].

GmXIPs are compared with 35 XIPs reported in [29] for NPA motif, intron/exon, P1-P5, and ar/R filters. The NPA motifs of GmXIP1;1 are similar to PtXIP2;1, while the 1^st^ motif of GmXIP1;2 is NPI as reported for PtXIP1;1, PtXIP1;2 and PtXIP1;3. Several XIPs from fungi contain one intron as in the present studies. The ar/R filter of GmXIP1;1 (VIVR) is similar to PtXIP2;1 and/or RcXIP2;3.

MIPs also transport non-aqua substrates such as ammonia, urea etc [25]. GmMIPs specificity for non-aqua substrates was detected *in silico*. Members of GmPIPs may facilitate transport of B, CO_2_, H_2_O_2_ and urea, while those of GmTIPs may transport H_2_O_2_ and urea (Fig. S5). The SDPs analysis revealed that GmNIP2s (subgroup III) may work as H_2_O_2_ transporters, GmNIP5;1 (subgroup IIA) as both H_2_O_2_ and urea transporter, however none of the GmMIPs are silicic acid transporters. GmNIP6;1 (subgroup IVA) and GmNIP6;2 (subgroup IVB) have SDPs for B and urea while GmNIP6;2 may also transport H_2_O_2_. Some members of the GmNIPs may also transport ammonia as well. SDPs analysis further predicted that ammonia and CO_2_ transport is a specific characteristic of GmNIPs and GmPIPs, respectively. Such specific characteristics of subfamilies has also been predicted in plant MIPs [25].

### Expression analysis of GmMIPs

Specific tools such as cDNA hybridization, fine mapping based on *in situ* RT-PCR, qPCR etc. have recently been used to monitor expression of the whole aquaporin gene family. Hybridization of cDNAs to arrays carrying aquaporin gene specific tags have revealed a coordinated down-regulation of aquaporin genes in response to water and nutrient stresses [11], [19]. Quantitative PCR analyses have been used to monitor expression of aquaporin transcripts in various tissues, organs or stress conditions [11], [14], [19]. In the present study, we used semi-qPCR and qPCR analyses to establish the relationship of specific MIP abundance and drought tolerance in soybean roots. Among 24 soybean aquaporin genes selected on the basis of *in silico* expression in roots and phylogenetic relationship, our results indicated that at least ten genes expressed in roots and the expression of four genes encoding PIP and three genes encoding TIP was significantly greater than the internal control (Fig. 5). Most of these TIPs and PIPs are located on chromosomes 11 and 12. In Arabidopsis, a similar pattern has been observed in roots, leaves and flowers, where three PIPs, five TIPs, seven NIPs and one SIPs were expressed in roots [19]. Orthologous MIPs in rice also expressed in roots, however the expression of OsTIP, OsNIP1;1, OsNIP1;4, OsNIP2;2, OsNIP3;3, OsNIP4;1 and OsSIP2;1 was weaker than the control [14]. This study showed organ specificity of GmMIPs as reported earlier in Arabidopsis [19] and rice [14].

The genes expressing in roots and those which are located on chromosomes 11 and 12 were selected for subsequent testing water stress response. GmPIP2;4 didn't express in any organ (Fig. 5) until 21 d after cessation of watering (Fig. 6). However, it expressed in roots 12 h after growth in 20% PEG (Fig. 7). GmTIP1;7 also didn't express in roots (Fig. 5) until 7 d without watering (Fig. 6). Such expression patterns were also reported for OsTIP4;2 in rice, where its expression is very low in roots growing in normal conditions and significantly increased in roots after 10 h growth in 15% PEG and 8 h growth in 150 mM NaCl stress [43]. It can be speculated that GmPIP2;4 and GmTIP1;7 are responsive to water stress as they only expressed after a period of water stress and are thus putatively useful. The qPCR analysis of nine GmPIPs and five TIPs further established the relationship of these GmMIPs with drought tolerance (Fig. 7). Three patterns were evident, down-regulation followed by up-regulation of GmMIPs and vice versa at different times after initiation of drought stress. The third type of expression pattern was revealed by qPCR analysis of GmPIP2;5 and GmTIP2;6. The former did not express in any plant organ (Fig. 5) but expressed after 12 h of drought stress in root (Fig. 7), while the later didn't express in roots (Fig. 5) but expressed 2 and 4 h after application of drought stress (Fig. 7). Similar expression patterns of various genes have been studied in Arabidopsis for urea transport [44] and for drought [19], and in rice for chilling and light reception [14] and for drought and salinity stress [43]. The expression pattern of GmMIPs after application of drought treatment reflected a coordinated regulation of MIP isoforms that collectively contribute to the whole root water transport capacity. These GmMIPs are a potential resource for the genetic improvement of soybean drought tolerance.

### Conclusions

This study identified and characterized 66 soybean aquaporin genes. Phylogenetic analysis of amino acid sequences divided the large and highly similar multi-gene family into 5 subfamilies. These genes were further classified into twelve subgroups: GmPIPs located in one subgroup, GmTIPs in three, GmNIPs in four, GmSIPs in three and GmXIPs in one. It can be speculated that GmMIPs contains true aquaporins, glyceroporins, aquaglyceroporins and mixed transport facilitators. However, their functionality remains to be properly validated. Our results indicate that the genes identified in this study represent an important genetic resource for the improvement of water use efficiency and/or drought tolerance as well as for transport of non-aqua substrate in soybean.

## Supporting Information

Table S1 **The primer sequences, positions and expected product sizes used in semi-qPCR and qPCR.**
(DOCX)

Table S2 **Ka/ks values of 64 GmAQPs.**
(DOCX)

Figure S1 **Phylogenetic analysis of 66** ***Gm*** **MIPs with those of moss, Arabidopsis, rice and maize to establish nomenclature to unknown genes.**
(TIF)

Figure S2 **Phylogenetic analysis of 66 GmMIPs with bootstrap test.**
(TIF)

Figure S3 **ka/ks annotated evolutionary tree of 64** ***Gm*** **MIPs.**
(PDF)

Figure S4 **Exon/Intron analysis of 66 GmMIPs. Exon 1 is shown by yellow color, 2 by green, 3 by blue, 4 by red, 5 by pink and 6 by dark green.**
(TIFF)

Figure S5 **Alignments of putative amino acids sequences of GmMIPs transporting non-aqua substrates.**
(DOC)
