# Correction: Genome-Wide Sequence Characterization and Expression Analysis of Major Intrinsic Proteins in Soybean (*Glycine max* L.)

**DOI:** 10.1371/annotation/e3307d0c-bb59-4f75-89b4-8e0d5af087d5

**Published:** 2014-01-16

**Authors:** Da Yong Zhang, Zulfiqar Ali, Chang Biao Wang, Ling Xu, Jin Xin Yi, Zhao Long Xu, Xiao Qing Liu, Xiao Lan He, Yi Hong Huang, Iqrar Ahmad Khan, Richard M. Trethowan, Hong Xiang Ma

The affiliation of the fifth author, Dr. Igrar Ahmad Kahn, is incorrect and should be Centre of Agricultural Biochemistry and Biotechnology (CABB), University of Agriculture, Faisalabad, Pakistan. 

Additionally, Dr. Jin Xin Yi has been added as a third corresponding author. Dr. Yi can be contacted at YIJ2111@gmail.com. 

