# Supplementary material for: Genome-Wide Sequence Characterization and Expression Analysis of Major Intrinsic Proteins in Soybean (Glycine max L.)
Source: PLoS One. 2013 Feb 20;8(2):e56312. doi: 10.1371/journal.pone.0056312 (PMC3577755; doi:10.1371/journal.pone.0056312)
Supplement: Table S1 — The primer sequences, positions and expected product sizes used in semi-qPCR and qPCR. (DOCX) [file pone.0056312.s001.docx]

Table S1. The primer sequences, positions and expected product sizes used in semi-qPCR and qPCR.

| Gene | Forward primer 5́-3́ | Reverse primer 5́-3́ | Strat position | End position | Product size (bp) |
| --- | --- | --- | --- | --- | --- |
| GmPIP1;3 | CTTATTTCGCAGAGTTCCAC | AACCCAGTAGATCCAGTTAGC | 58 | 653 | 596 |
| GmPIP1;4 | CACAGTGGTCCTTTTATAGAGC | GCAGAGAAGACAGTGTAGACAA | 100 | 565 | 466 |
| GmPIP1;7 | CTAAGTGTAGTACCGTTGGGAT | ATGATAGCAGCACCAAGACTAC | 247 | 745 | 499 |
| GmPIP1;8 | ACTCACTTCTTGGTCCTTCTAC | AATAGGGACGTGTGAGTCTCT | 140 | 638 | 499 |
| GmPIP2;1 | CATGATCTTTGTCCTCGTCTAC | CTCTCAGGATGTACTGATGGTA | 230 | 777 | 548 |
| GmPIP2;3 | CTCTGTTGGTGCTAACATCTC | CCCAGTAGACCCAGTGATTA | 218 | 660 | 443 |
| GmPIP2;4 | CGTTCATAGTCATAGTCGTGAG | CTCCATCACACTCTTGAGTACA | 20 | 437 | 418 |
| GmPIP2;5 | CCTAAACTCCATCTATGTCCTC | GTGTTGTGCTTGTTGTTGAC | 149 | 610 | 462 |
| GmPIP2;6 | CTGTAAGCACTAACATCTCAGG | GGCTATGGCTAACAAAGATG | 211 | 723 | 513 |
| GmPIP2;10 | ACTCACACAGTGGTCCTTCTAC | CAGAGAACACAGTGTAGACCAA | 86 | 555 | 470 |
| GmPIP2;11 | GTTACAAGAGCCAGAGTGATG | TAGACTCCTAGCAGGGTTAATG | 175 | 692 | 518 |
| GmPIP2;13 | CCGTCCTTAGAGCAGTTTACTA | GTATTCATACAGGAGTGCTGC | 295 | 707 | 413 |
| GmTIP1;2 | GTCTCATTGCTGCATCACTATC | ACCAACTAAGATGTTGGCAC | 169 | 569 | 401 |
| GmTIP1;4 | CCTCTCTAATGTTCACCTTGAC | CCCAGTTACAGTTATGGACAC | 37 | 563 | 527 |
| GmTIP1;7 | GGCAACATCACCTTACTCC | GAAGACAACCTCGTAGATAAGC | 285 | 710 | 426 |
| GmTIP1;8 | CTCAACCTTCATCTTCGTGT | CAGTAGATCCAGTGGTTGGT | 83 | 655 | 573 |
| GmTIP1;9 | TACAAGAGACAGAGTGACACCA | CTAACACAGGAACATGAGAGTC | 186 | 615 | 430 |
| GmTIP2;1 | AAGAGTGACAAGGACTACAAGG | AGAGTCTCTAGCGTTTCTCTTG | 72 | 614 | 543 |
| GmTIP2;2 | CTTGTTCCTCTACATCACTGTC | AGAGTCTCTAGCGTTTCTCTTG | 182 | 614 | 433 |
| GmTIP2;6 | ACTGGTCTCTTCTACTGGATTG | GGGATGAAGGCATAGGTATAG | 297 | 709 | 413 |
| GmTIP4;1 | GGTGGTCACATCACTATCTTTC | CTCATAGATGTAACCAGCAAGG | 264 | 686 | 423 |
| GmNIP6;2 | AGAGTTGTAGGTGCTTCAGTGT | GCCCTGCTATGAGTATATTGAG | 115 | 714 | 600 |
| GmSIP1;3 | ATGGTTGGTGCTATAAAAGCAGCGA | TCATGCTTTCTTCTGTTTTACTTC | 1 | 747 | 747 |
| GmXIP1;2 | GGTTACAGAGCAAGGTGAATAC | CCGTACCTGTTGTAGTACGATT | 20 | 469 | 450 |
