# Supplementary material for: Genome-Wide Sequence Characterization and Expression Analysis of Major Intrinsic Proteins in Soybean (Glycine max L.)
Source: PLoS One. 2013 Feb 20;8(2):e56312. doi: 10.1371/journal.pone.0056312 (PMC3577755; doi:10.1371/journal.pone.0056312)
Supplement: Table S2 — Ka/ks values of 64 GmAQPs. (DOCX) [file pone.0056312.s002.docx]

Table S2. Ka/ks values of 64 GmMIPs.

| Node# | Ka/Ks Branch1 | **Ka Branch1** | **Ks Branch1** | **Ka/Ks Branch2** | **Ka Branch2** | **Ks Branch2** |
| --- | --- | --- | --- | --- | --- | --- |
| 1 | 0.15 | 0.01 | 0.09 | 0.07 | 0.00 | 0.07 |
| 2 | 0.25 | 0.03 | 0.14 | 1.12 | 0.18 | 0.16 |
| 3 | 0.03 | 0.00 | 0.08 | 0.09 | 0.01 | 0.06 |
| 4 | 0.09 | 0.02 | 0.26 | 1.60 | 0.65 | 0.41 |
| 5 | 0.24 | 0.10 | 0.40 | 0.13 | 0.07 | 0.55 |
| 6 | 0.11 | 0.00 | 0.04 | 0.07 | 0.00 | 0.04 |
| 7 | 0.04 | 0.00 | 0.04 | 0.09 | 0.00 | 0.05 |
| 8 | 0.13 | 0.05 | 0.40 | 0.11 | 0.03 | 0.26 |
| 9 | 0.09 | 0.02 | 0.20 | 0.04 | 0.01 | 0.26 |
| 10 | 0.41 | 0.03 | 0.07 | 0.10 | 0.01 | 0.09 |
| 11 | 0.08 | 0.03 | 0.32 | 0.08 | 0.03 | 0.34 |
| 12 | 0.12 | 0.04 | 0.29 | 0.19 | 0.06 | 0.30 |
| 13 | 0.08 | 0.00 | 0.05 | 0.19 | 0.01 | 0.04 |
| 14 | 0.29 | 0.00 | 0.01 | 0.23 | 0.00 | 0.01 |
| 15 | 0.16 | 0.01 | 0.05 | 0.00 | 0.00 | 0.02 |
| 16 | 0.00 | 0.00 | 0.07 | 0.00 | 0.00 | 0.06 |
| 17 | 0.14 | 0.02 | 0.17 | 0.20 | 0.03 | 0.15 |
| 18 | 0.13 | 0.01 | 0.04 | 0.10 | 0.00 | 0.04 |
| 19 | 0.10 | 0.01 | 0.12 | 0.17 | 0.01 | 0.07 |
| 20 | 0.15 | 0.04 | 0.26 | 0.23 | 0.12 | 0.54 |
| 21 | 0.16 | 0.00 | 0.03 | 0.06 | 0.00 | 0.04 |
| 22 | 0.13 | 0.03 | 0.25 | 0.13 | 0.02 | 0.19 |
| 23 | 0.14 | 0.04 | 0.27 | 0.14 | 0.04 | 0.31 |
| 24 | 0.20 | 0.05 | 0.26 | 0.21 | 0.06 | 0.27 |
| 25 | 0.41 | 0.08 | 0.20 | 0.32 | 0.06 | 0.20 |
| 26 | 0.87 | 0.02 | 0.03 | 0.30 | 0.01 | 0.04 |
| 27 | 0.80 | 0.39 | 0.48 | 0.78 | 0.34 | 0.44 |
| 28 | 0.49 | 0.25 | 0.51 | 0.26 | 0.07 | 0.26 |
| 29 | 0.06 | 0.00 | 0.08 | 0.14 | 0.01 | 0.06 |
| 30 | 0.16 | 0.05 | 0.33 | 0.26 | 0.08 | 0.29 |
| 31 | 0.49 | 0.18 | 0.36 | 0.76 | 0.30 | 0.40 |
| 32 | 0.13 | 0.01 | 0.07 | 0.26 | 0.01 | 0.05 |
| 33 | 0.48 | 0.23 | 0.49 | 1.00 | 0.55 | 0.55 |
| 34 | 0.20 | 0.06 | 0.31 | 0.27 | 0.08 | 0.31 |
| 35 | 0.54 | 0.26 | 0.49 | 0.44 | 0.16 | 0.36 |
| 36 | 0.26 | 0.01 | 0.05 | 0.39 | 0.02 | 0.04 |
| 37 | 0.15 | 0.06 | 0.38 | 0.28 | 0.10 | 0.35 |
| 38 | 0.26 | 0.10 | 0.38 | 0.34 | 0.13 | 0.39 |
| 39 | 0.56 | 0.28 | 0.50 | 0.60 | 0.21 | 0.35 |
| 40 | 0.81 | 0.26 | 0.32 | 0.39 | 0.16 | 0.41 |
| 41 | 0.50 | 0.01 | 0.02 | 0.00 | 0.00 | 0.01 |
| 42 | 0.09 | 0.00 | 0.03 | 0.09 | 0.00 | 0.04 |
| 43 | 0.02 | 0.00 | 0.04 | 0.18 | 0.00 | 0.01 |
| 44 | 0.17 | 0.03 | 0.15 | 0.21 | 0.03 | 0.15 |
| 45 | 0.36 | 0.03 | 0.07 | 0.46 | 0.05 | 0.10 |
| 46 | 0.16 | 0.01 | 0.06 | 0.25 | 0.01 | 0.05 |
| 47 | 0.77 | 0.35 | 0.45 | 0.19 | 0.10 | 0.50 |
| 48 | 0.21 | 0.05 | 0.25 | 0.23 | 0.07 | 0.30 |
| 49 | 0.17 | 0.01 | 0.05 | 0.11 | 0.00 | 0.04 |
| 50 | 0.06 | 0.00 | 0.05 | 0.18 | 0.01 | 0.05 |
| 51 | 0.37 | 0.07 | 0.20 | 0.29 | 0.06 | 0.21 |
| 52 | 0.07 | 0.01 | 0.09 | 0.10 | 0.01 | 0.08 |
| 53 | 0.07 | 0.00 | 0.07 | 0.14 | 0.01 | 0.05 |
| 54 | 0.51 | 0.25 | 0.49 | 0.37 | 0.11 | 0.31 |
| 55 | 0.23 | 0.07 | 0.31 | 0.27 | 0.08 | 0.29 |
| 56 | 0.48 | 0.16 | 0.34 | 0.13 | 0.07 | 0.53 |
| 57 | 0.13 | 0.04 | 0.31 | 0.07 | 0.01 | 0.11 |
| 58 | 0.10 | 0.01 | 0.07 | 0.20 | 0.02 | 0.08 |
| 59 | 0.32 | 0.01 | 0.04 | 0.09 | 0.00 | 0.04 |
| 60 | 0.31 | 0.05 | 0.16 | 0.09 | 0.02 | 0.19 |
| 61 | 0.21 | 0.06 | 0.27 | 0.16 | 0.06 | 0.34 |
| 62 | 0.09 | 0.03 | 0.30 | 0.26 | 0.08 | 0.29 |
| 63 | 0.36 | 0.10 | 0.27 | 0.23 | 0.07 | 0.31 |
| 64 | 0.47 | 0.18 | 0.38 | 0.30 | 0.13 | 0.42 |
