# Supplementary figures and images for: Genome-Wide Sequence Characterization and Expression Analysis of Major Intrinsic Proteins in Soybean (Glycine max L.)
Source: PLoS One. 2013 Feb 20;8(2):e56312. doi: 10.1371/journal.pone.0056312 (PMC3577755; doi:10.1371/journal.pone.0056312)

**Ka/Ks annotated evolutionary tree**

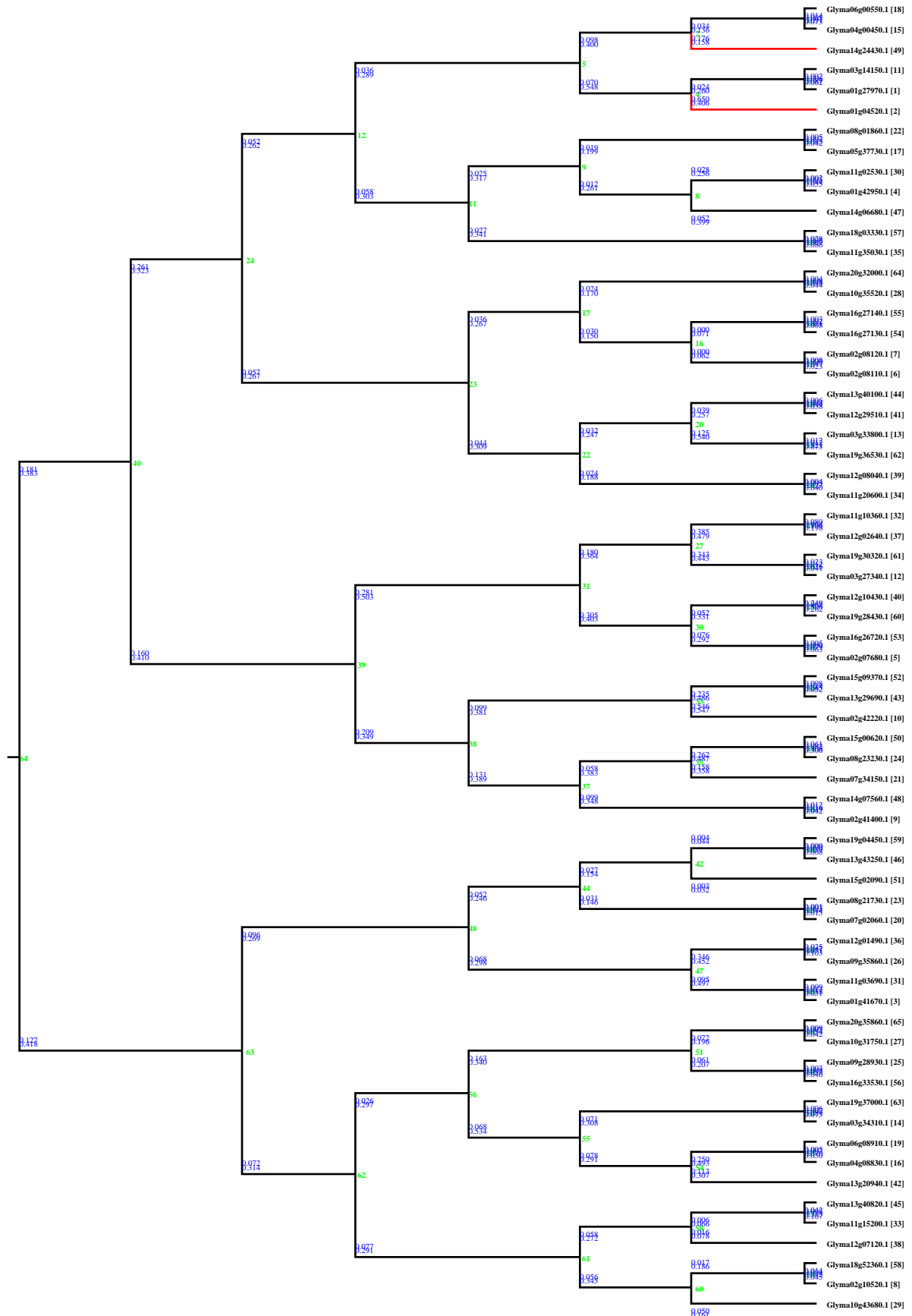

Supplement: Figure S3 — ka/ks annotated evolutionary tree of 64 Gm MIPs. (PDF) [file pone.0056312.s005.pdf]

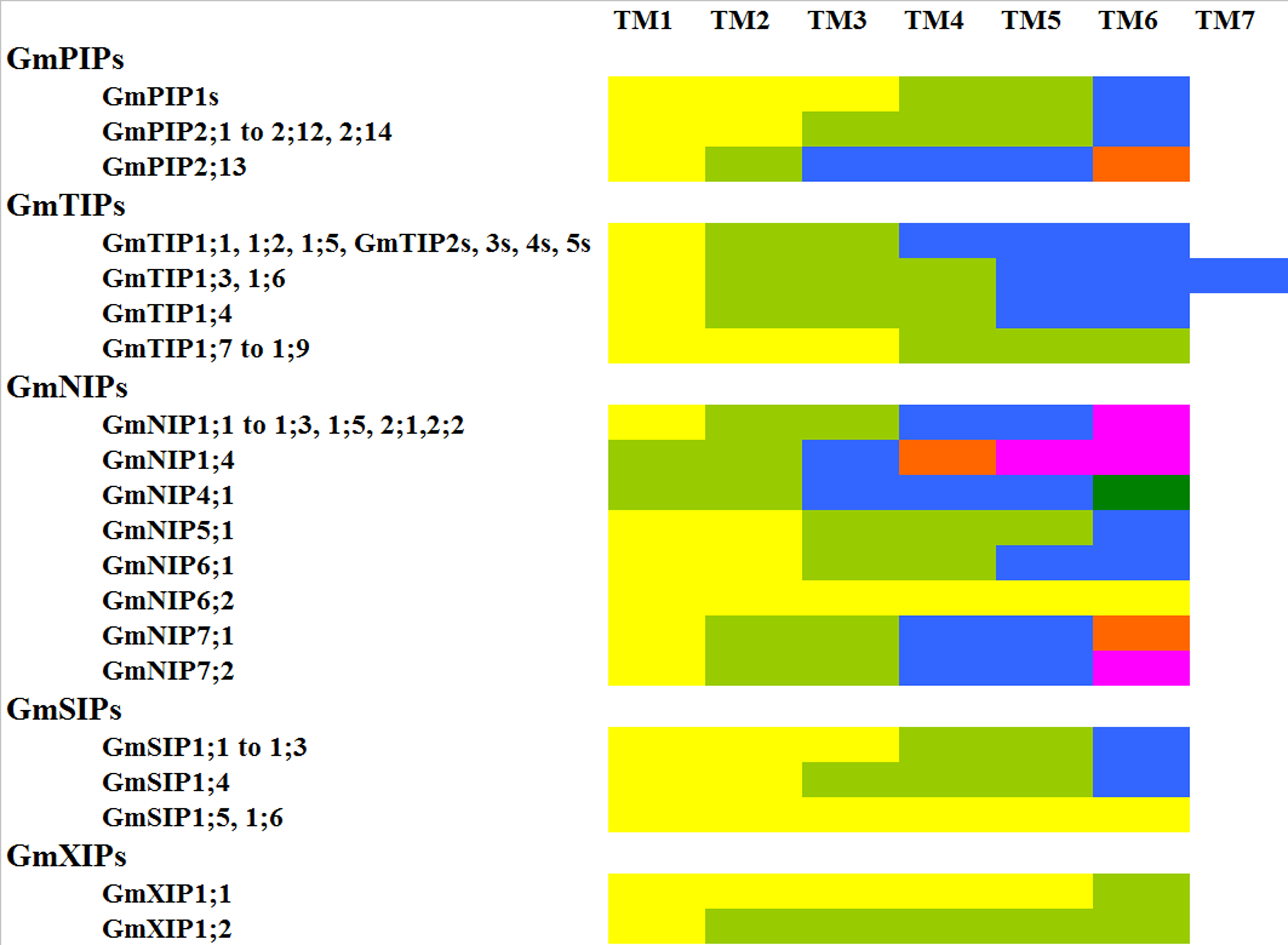

Supplement: Figure S4 — Exon/Intron analysis of 66 GmMIPs. Exon 1 is shown by yellow color, 2 by green, 3 by blue, 4 by red, 5 by pink and 6 by dark green. (TIFF) [file pone.0056312.s006.tif]
