# Supplementary material for: Genome-Wide Sequence Characterization and Expression Analysis of Major Intrinsic Proteins in Soybean (Glycine max L.)
Source: PLoS One. 2013 Feb 20;8(2):e56312. doi: 10.1371/journal.pone.0056312 (PMC3577755; doi:10.1371/journal.pone.0056312)
Supplement: Figure S5 — Alignments of putative amino acids sequences of GmMIPs transporting non-aqua substrates. (DOC) [file pone.0056312.s007.doc]

**Ammonia**

**TM1**

GmNIP1.1 MDENSATNGTHEVVLDVNRDVSRTTQASRSCVNVSFLQKLVAEVVGTYFLIFAGSASVVV 60(60)

GmNIP1.2 MDENSATNGTHEVILDVNKDVSRTTQPSRSCVNVSFLQKLVAEVVGTYFLIFAGCASVVV 60(60)

GmNIP1.5Nod26 MADYSAGTESQEVVVNVTKNTSETIQRSDSLVSVPFLQKLVAEAVGTYFLIFAGCASLVV 60(60)

* : ** . ::**:::*.::.*.* * * * *.*.********.**********.**:**

**TM2\**

**HB**

**TM3**

GmNIP1.1 NKNNNNVVTLPGISIVWGLVVMVLVYSVGHISGAHFNPAVTIAFASTKRFPLKQVPVYVV 120(120)

GmNIP1.2 NKNNNNVVTHPGISIVWGLVVMVLVYSVGHISGAHFNPAVTIAFASTRRFPLKQVPVYVV 120(120)

GmNIP1.5Nod26 NENYYNMITFPGIAIVWGLVLTVLVYTVGHISGGHFNPAVTIAFASTRRFPLIQVPAYVV 120(120)

*:* *::* ***:******: ****:******.*************:**** ***.***

**TM4**

**TM3**

GmNIP1.1 AQVVGSTLASGTLRLLFSGKEAQFSGTLPSGSNLQAFVIEFLITFFLMFVVSGVATDNRA 180(180)

GmNIP1.2 AQVVGSTLASATLRLLFSGKETQFSGTLPSGSNLQAFVIEFLITFFLMFVISGVATDDRA 180(180)

GmNIP1.5Nod26 AQLLGSILASGTLRLLFMGNHDQFSGTVPNGTNLQAFVFEFIMTFFLMFVICGVATDNRA 180(180)

**::** ***.****** *:. *****:*.*:******:**::*******:.*****:**

**TM5**

**HE**

**TM6**

GmNIP1.1 IGELAGIAVGSTVLLNVMFAGPITGASMNPARSIGPAIVHKEYRGIWIYLVSPTLGAVAG 240(240)

GmNIP1.2 IGELAGIAVGSTVLLNVMFAGPITGASMNPARSIGPAILHNEYRGIWIYIVSPTLGAVAG 240(240)

GmNIP1.5Nod26 VGELAGIAIGSTLLLNVIIGGPVTGASMNPARSLGPAFVHGEYEGIWIYLLAPVVGAIAG 240(240)

:*******:***:****::.**:**********:***::* **.*****:::*.:**:**

**TM6**

GmNIP1.1 AWVYNSIRYTDKPLREITKSASFLKGVASR---- 270(274)

GmNIP1.2 TWVYNTIRYTDKPLREITKSTSFLKGVGRSGSSR 274(274)

GmNIP1.5Nod26 AWVYNIVRYTDKPLSEITKSASFLKGRAASK--- 271(274)

:**** :******* *****:***** .

**Borric acid**

GmPIP1.7 -------------------------MEGKEQDVSLGANKFPERQPIGTAAQSQDDGKDYQ 35(60)

GmPIP1.8 -------------------------MEGKEEDVSLGANKFSERQPIGTAAQSQDDGKDYT 35(60)

GmPIP1.1 -------------------------MEGKEEDVRVGANRYGERQPIGTAAQ----AKDYR 31(60)

GmPIP1.2 --------LRLPHSHIHIEVKLALKMEGRDEDVRVGANRYGERQPIGTAAQTQD-AKDYR 51(60)

GmPIP1.3 --------------------------MEREEDVKVGAQKFSERQALGTGAKS---DKDYK 31(60)

GmPIP1.4 --------------------------MEREEDVKVGAQKFSERQALGTGAQG---DKDYK 31(60)

GmPIP1.5 -------------------------MESKEEDVRVGATKFSERQPIGTAAQG---DKDYK 32(60)

GmPIP1.6 -------------------------MESKEEDVNVGANKFSERQPIGTAAQGGG-DKDYK 34(60)

GmPIP2.7 -----------------------------------------MAKDIETEVQSGLPHKDYH 19(60)

GmNIP6.2 -MDNNEEIPSTP----ATPGTPGAPLFGGFSNGRNNNS----KKSLLKSCRCFSVE-EWS 59(60)

GmNIP6.1 --MNNEEVPSLPSTS-ATPGTPGAPLFGGLRFEKPNGS-VVRKSSFLKSCKCFSVA-EWT 58(60)

AtNIP5_1 MAPPEAEVGAVMVMAPPTPGTPGTPGGPLITGMRVDSMSFDHRKPTPRCKCLPVMGSTWG 60(60)

. :

**TM1**

GmPIP1.7 EPAPAPLVDPTEFTSWS------FYRAGIAEFVATFLFLYITVLTVMGVAGAKSK----- 84(120)

GmPIP1.8 EPPPAPLFEPSELTSWS------FYRAGIAEFVATFLFLYITILTVMGVNRSSSK----- 84(120)

GmPIP1.1 EPPSAPLFEPGELSSWS------FYRAGIAEFVATFLFLYITVLTVMGVFKSKSK----- 80(120)

GmPIP1.2 EAPPAPLFEPRELTSWS------FYRAGIAEFVATFLFLYVTVLTVMGVAKSPSK----- 100(120)

GmPIP1.3 EAPPAPLFEPGELKSWS------FYRAGIAEFVATFLFLYITVLTVMGVNRAPNK----- 80(120)

GmPIP1.4 EAPPAPLFEPGELKSWS------FYRAGIAEFVATFLFLYITVLTVMGVNRAPNK----- 80(120)

GmPIP1.5 EPPPAPLFEPGELKSWS------FYRAGIAEFVATFLFLYITILTVMGVNRSPSK----- 81(120)

GmPIP1.6 EAPPAPLFEPGELKSWS------FYRAGIAEFVATFLFLYITILTVMGVNRSPSK----- 83(120)

GmPIP2.7 DPPAAAFYDPAELRKWS------FYRALIAEFVATLLFLYVTILTVIGYNHQTATGSPDL 73(120)

GmNIP6.2 LEDGGLPAVSCSLPLPSPPPVVPLARKIGAEFIGTFILMFAGTAAAIVNQKTNGS----- 114(120)

GmNIP6.1 LEDGAMPRVSCSLPSP----HIPLAKKIGAEFIGTFILMFAAIGTAIVNQKTHGS----- 109(120)

AtNIP5_1 QHDTCFTDFPSPD--------VSLTRKLGAEFVGTFILIFTATAGPIVNQKYDGA----- 107(120)

. : : ***:.*::::: :

**TM3**

**TM2**

**HB**

GmPIP1.7 CSTVGIQGIAWAFGGMIFALVYCTAGISGGHINPAVTFGLFLARKLSLPRAIFYIVMQCL 144(180)

GmPIP1.8 CATVGIQGIAWAFGGMIFALVYCTAGISGGHINPAVTFGLFLARKLSLTRALFYMVMQVL 144(180)

GmPIP1.1 CSTVGIQGIAWAFGGMIFALVYSTAGISGGHINPAVTFGLFLARKLSLTRAIFYIIMQCL 140(180)

GmPIP1.2 CSTVGVQGIAWSFGGMIFALVYCTAGISGGHINPAVTFGLFLARKLSLTRTVFYMIMQCL 160(180)

GmPIP1.3 CSSVGIQGIAWAFGGMIFALVYCTAGISGGHINPAVTFGLFLARKLSLTRAVFYIVMQCL 140(180)

GmPIP1.4 CSSVGIQGIAWAFGGMIFALVDCTAGISGGHINPAVTFGLFLARKLSLTRALFYIVMQCL 140(180)

GmPIP1.5 CASVGIQGIAWAFGGMIFALVYCTAGISGGHINPAVTFGLFLARKLSLTRALFYIIMQCL 141(180)

GmPIP1.6 CASVGIQGIAWAFGGMIFALVYCTAGISGGHINPAVTFGLFLARKLSLTRALFYIIMQCL 143(180)

GmPIP2.7 CNGVGVLGIAWAFGGMIFVLVYCTAGISGGHINPAVTFGLFLARKVSLIRAVGYMVAQVL 133(180)

GmNIP6.2 ---ETLIGCAATTGLAVMIVILATGHISGAHLNPAVTISFAALKHFPWKHVPMYIGAQVL 171(180)

GmNIP6.1 ---ETLIGCAAANGLAVMIIIFSTGHISGAHLNPAVTISFAALKHFPWKNVPVYIGTQVL 166(180)

AtNIP5_1 ---ETLIGNAACAGLAVMIIILSTGHISGAHLNPSLTIAFAALRHFPWAHVPAYIAAQVS 164(180)

: * * * :: :: .*. ***.*:**::*:.: ::.. .. *: *

**TM3**

**TM4**

GmPIP1.7 GAICGAGVVKGFEGKTKYGALNGGANFVAPGYTKGDGLGAEIVGTFILVYTVFSATDAKR 204(240)

GmPIP1.8 GAIVGAGVVKGFEGKTFYGQHNGGANFVAPGYTKGDGLGAEIVGTFILVYTVFSATDAKR 204(240)

GmPIP1.1 GAICGAGVVKGFEPHL-YERLGGGANTIAKGYTNSAGLGAEIVGTFVLVYTVFSATDAKR 200(240)

GmPIP1.2 GAICGAAVVKGFQSNQ-YERLGGGANTLSKGYSKGDGLGAEIVGTFILVYTVFSATDAKR 220(240)

GmPIP1.3 GAICGAGVVKGFEGNARYELFKGGANFVSHGYTKGDGLGAEIVGTFILVYTVFSATDAKR 200(240)

GmPIP1.4 GAICGAGVVKGFEGNARYELFKGGANFVSHGYTKGDGLGAEIVGTFILVYTVFSATDAKR 200(240)

GmPIP1.5 GAICGAGVVKGFEGNARYEMFKGGANFVNSGYTKGDGLGAEIVGTFVLVYTVFSATDAKR 201(240)

GmPIP1.6 GAICGAGVVKGFEGNANYELFKGGANFVNSGYTKGDGLGAEIVGTFVLVYTVFSATDAKR 203(240)

GmPIP2.7 GAISGVGLVKALQKS-YYNRYNGGVNMLADGYSKGTGLGAEIIGTFILVYTVFSATDPKR 193(240)

GmNIP6.2 ASICAGFALK----GVYHPFMSGGVTVPSGGYG--QSFALEFIIGFNLMFVVTAVATDTR 225(240)

GmNIP6.1 ASVSAAFALK----VVFHPFMSGGVTVPSVGYG--QAFATEFIVSFILMFVVTAVATDTR 220(240)

AtNIP5_1 ASICASFALK----GVFHPFMSGGVTIPSVSLG--QAFALEFIITFILLFVVTAVATDTR 218(240)

.:: . :* **.. . .:. *:: * *::.* :.: .*

**TM5**

**TM6**

**HE**

GmPIP1.7 SARDSHVPILAPLPIGFAVFLVHLATIPITGTGINPARSLGAAIIFNKDLGWDEHWIFWV 264(300)

GmPIP1.8 SARDSHVPILAPLPIGFAVFLVHLATIPITGTGINPARSLGAAIIFNKDLGWDDHWIFWV 264(300)

GmPIP1.1 NARDSHVPILAPLPIGFAVFLVHLATIPVTGTGINPARSLGAAIIFNKDQAWDDHWIFWV 260(300)

GmPIP1.2 NARDSHVPILAPLPIGFAVFLVHLATIPITGTGINPARSLGAALVYNKDQAWDNHWIFWV 280(300)

GmPIP1.3 NARDSHVPILAPLPIGFAVFLVHLATIPITGTGINPARSLGAAIIYNRDHAWDDHWIFWV 260(300)

GmPIP1.4 NARDSHVPILAPLPIGFAVFLVHLATIPITGTGINPARSLGAAIIYNRDHAWDDHWIFWV 260(300)

GmPIP1.5 NARDSHVPILAPLPIGFAVFLVHLATIPITGTGINPARSLGAAIIYNRDHAWDDQWIFWV 261(300)

GmPIP1.6 NARDSHVPILAPLPIGFAVFLVHLATIPITGTGINPARSLGAAIIYNRDHAWDDQWIFWV 263(300)

GmPIP2.7 VARDSHVPVLAPLPIGFAVFIVHLATIPITGTGINPARSLGPAVIFNNEKAWDDQWIFWV 253(300)

GmNIP6.2 -----AVGELAGIAVGATVMLNILIAGPVSGGSMNPVRTLGPAVAANN---YKAIWVYLV 277(300)

GmNIP6.1 -----AVGELAGIAVGATVMLNILIAGPTTGSSMNPVRTLGPAIAANN---YKGIWVYLI 272(300)

AtNIP5_1 -----AVGELAGIAVGATVMLNILVAGPSTGGSMNPVRTLGPAVASGN---YRSLWVYLV 270(300)

* ** :.:* :*:: * : * :. .:**.*:**.*: .. : *::

**TM6**

GmPIP1.7 GPFIGAALAALYHQVVIRAIPFKSK------------ 289(337)

GmPIP1.8 GPFVGAALAALYHQVVIRAIPFKSS------------ 289(337)

GmPIP1.1 GPFIGAALAALYHQIVIRAIPFSSK------------ 285(337)

GmPIP1.2 GPFIGAALAALYHQIVLRAIPFKSK------------ 305(337)

GmPIP1.3 GPFIGAALAAVYHQIVIRAIPFKTRG----------- 286(337)

GmPIP1.4 GPFIGAALAALYHQIVIRAIPFKTRG----------- 286(337)

GmPIP1.5 GPFIGAALAAVYHQIVIRAIPFKTRA----------- 287(337)

GmPIP1.6 GPFIGAALAAVYHQIVIRAIPFKTRA----------- 289(337)

GmPIP2.7 GPFIGAAIAAFYHQSVLRAQAAKALGSFRSSSNL--- 287(337)

GmNIP6.2 APILGALAGAGTYTAVKLPEEDDDAKAKTSISSFRR- 313(337)

GmNIP6.1 APILGTLCGAGAYTVVKLPEEEATKTPSSAPNGSFRR 309(337)

AtNIP5_1 APTLGAISGAAVYTGVKLNDSVTDPPRPVRSFRR--- 304(337)

.* :*: .* : *

**Carbon dioxide**

**TM1**

GmPIP1.5 MESKEEDVRVGATKFSERQPIGTAAQG--DKDYKEPPPAPLFEPGELKSWSFYRAGIAEF 58(60)

GmPIP1.6 MESKEEDVNVGANKFSERQPIGTAAQGGGDKDYKEAPPAPLFEPGELKSWSFYRAGIAEF 60(60)

GmPIP1.3 -MEREEDVKVGAQKFSERQALGTGAKS--DKDYKEAPPAPLFEPGELKSWSFYRAGIAEF 57(60)

GmPIP1.4 -MEREEDVKVGAQKFSERQALGTGAQG--DKDYKEAPPAPLFEPGELKSWSFYRAGIAEF 57(60)

AtPIP1_2 MEGKEEDVRVGANKFPERQPIGTSAQS--DKDYKEPPPAPLFEPGELASWSFWRAGIAEF 58(60)

GmPIP1.1 MEGKEEDVRVGANRYGERQPIGTAAQA---KDYREPPSAPLFEPGELSSWSFYRAGIAEF 57(60)

:****.*** :: ***.:**.*:. ***:*.*.********* ****:*******

**TM2**

**TM1**

**HB**

GmPIP1.5 VATFLFLYITILTVMGVNRSPSKCASVGIQGIAWAFGGMIFALVYCTAGISGGHINPAVT 118(120)

GmPIP1.6 VATFLFLYITILTVMGVNRSPSKCASVGIQGIAWAFGGMIFALVYCTAGISGGHINPAVT 120(120)

GmPIP1.3 VATFLFLYITVLTVMGVNRAPNKCSSVGIQGIAWAFGGMIFALVYCTAGISGGHINPAVT 117(120)

GmPIP1.4 VATFLFLYITVLTVMGVNRAPNKCSSVGIQGIAWAFGGMIFALVDCTAGISGGHINPAVT 117(120)

AtPIP1_2 IATFLFLYITVLTVMGVKRSPNMCASVGIQGIAWAFGGMIFALVYCTAGISGGHINPAVT 118(120)

GmPIP1.1 VATFLFLYITVLTVMGVFKSKSKCSTVGIQGIAWAFGGMIFALVYSTAGISGGHINPAVT 117(120)

:*********:****** :: . *::****************** .**************

**TM3**

**HB**

**TM4**

GmPIP1.5 FGLFLARKLSLTRALFYIIMQCLGAICGAGVVKGFEGNARYEMFKGGANFVNSGYTKGDG 178(180)

GmPIP1.6 FGLFLARKLSLTRALFYIIMQCLGAICGAGVVKGFEGNANYELFKGGANFVNSGYTKGDG 180(180)

GmPIP1.3 FGLFLARKLSLTRAVFYIVMQCLGAICGAGVVKGFEGNARYELFKGGANFVSHGYTKGDG 177(180)

GmPIP1.4 FGLFLARKLSLTRALFYIVMQCLGAICGAGVVKGFEGNARYELFKGGANFVSHGYTKGDG 177(180)

AtPIP1_2 FGLFLARKLSLTRAVYYIVMQCLGAICGAGVVKGFQ-PKQYQALGGGANTIAHGYTKGSG 177(180)

GmPIP1.1 FGLFLARKLSLTRAIFYIIMQCLGAICGAGVVKGFE-PHLYERLGGGANTIAKGYTNSAG 176(180)

**************::**:****************: *: : **** : ***:. *

**TM5**

**TM4**

**HE**

GmPIP1.5 LGAEIVGTFVLVYTVFSATDAKRNARDSHVPILAPLPIGFAVFLVHLATIPITGTGINPA 238(240)

GmPIP1.6 LGAEIVGTFVLVYTVFSATDAKRNARDSHVPILAPLPIGFAVFLVHLATIPITGTGINPA 240(240)

GmPIP1.3 LGAEIVGTFILVYTVFSATDAKRNARDSHVPILAPLPIGFAVFLVHLATIPITGTGINPA 237(240)

GmPIP1.4 LGAEIVGTFILVYTVFSATDAKRNARDSHVPILAPLPIGFAVFLVHLATIPITGTGINPA 237(240)

AtPIP1_2 LGAEIIGTFVLVYTVFSATDAKRNARDSHVPILAPLPIGFAVFLVHLATIPITGTGINPA 237(240)

GmPIP1.1 LGAEIVGTFVLVYTVFSATDAKRNARDSHVPILAPLPIGFAVFLVHLATIPVTGTGINPA 236(240)

*****:***:*****************************************:********

**TM6**

**HE**

GmPIP1.5 RSLGAAIIYNRDHAWDDQWIFWVGPFIGAALAAVYHQIVIRAIPFKTRA 288(290)

GmPIP1.6 RSLGAAIIYNRDHAWDDQWIFWVGPFIGAALAAVYHQIVIRAIPFKTRA 290(290)

GmPIP1.3 RSLGAAIIYNRDHAWDDHWIFWVGPFIGAALAAVYHQIVIRAIPFKTRG 287(290)

GmPIP1.4 RSLGAAIIYNRDHAWDDHWIFWVGPFIGAALAALYHQIVIRAIPFKTRG 287(290)

AtPIP1_2 RSLGAAIIFNKDNAWDDHWVFWVGPFIGAALAALYHVIVIRAIPFKSRS 287(290)

GmPIP1.1 RSLGAAIIFNKDQAWDDHWIFWVGPFIGAALAALYHQIVIRAIPFSSK- 285(290)

********:*:*:****:*:*************:** ********.::

**Hydrogen peroxide**

AtTIP1.1 ---------------------------------------------------MPIRNIAIG 09(60)

GmPIP1.1 -----------------MEGKEEDVRVGANRYGERQPIGTAAQ----AKDYREPPSAPLF 39(60)

GmPIP1.2 LRLPHSHIHIEVKLALKMEGRDEDVRVGANRYGERQPIGTAAQTQD-AKDYREAPPAPLF 59(60)

GmPIP1.3 ------------------MEREEDVKVGAQKFSERQALGTGAKS---DKDYKEAPPAPLF 39(60)

GmPIP1.4 ------------------MEREEDVKVGAQKFSERQALGTGAQG---DKDYKEAPPAPLF 39(60)

GmPIP1.5 -----------------MESKEEDVRVGATKFSERQPIGTAAQG---DKDYKEPPPAPLF 40(60)

GmPIP1.6 -----------------MESKEEDVNVGANKFSERQPIGTAAQGGG-DKDYKEAPPAPLF 39(60)

GmPIP1.7 -----------------MEGKEQDVSLGANKFPERQPIGTAAQSQDDGKDYQEPAPAPLV 43(60)

GmPIP1.8 -----------------MEGKEEDVSLGANKFSERQPIGTAAQSQDDGKDYTEPPPAPLF 43(60)

GmPIP2.1 ------------------------------------MSKEVSQ---QRKDYVDPPPAPLI 21(60)

GmPIP2.2 ------------------------------------MSKEVSQEGLQRKDYVDPPPAPLF 24(60)

GmPIP2.7 ---------------------------------MAKDIETEVQSGLPHKDYHDPPAAAFY 27(60)

GmPIP2.8 ---------------------------------MAKDLETEIQSGLPHKDYHDPPPAPFY 27(60)

GmPIP2.9 -----------------------------------MAKHDVEGGSFSAKDYHDPPPAPLI 25(60)

GmPIP2.10 -----------------------------------MAKHDVEGGSFSAKDYHDPPPAPLI 25(60)

GmPIP2.11 -----------------------------------MAKHDVEGGSFAAKDYHDPPPAPLI 25(60)

GmPIP2.12 -----------------------------------MAKHDVEGGSFSAKDYHDPPPAPLI 25(60)

GmPIP2.13 ------------------------MSVFWQEGGMAKDVEVAERGSFSGKDYQDPPPAPLI 36(60)

GmPIP2.14 ---------------------------------MAKDVEVAERGSFSGKDYQDPPPAPLI 27(60)

GmTIP1.1 ---------------------------------------------------MAIYRIAIG 09(60)

GmTIP1.2 ---------------------------------------------------MAMHRIAIG 09(60)

GmTIP1.4 ---------------------------------------------------MPISRIAIG 09(60)

GmTIP1.5 ---------------------------------------------------MPISRIAIG 09(60)

GmTIP1.6 ---------------------------------------------------MPISRIAIG 09(60)

GmTIP1.7 ---------------------------------------------------MPIRNIAIG 09(60)

GmTIP1.8 ---------------------------------------------------MPIRNIAVG 09(60)

GmTIP3.1 ---------------------------------------------------MATRRYAFG 09(60)

GmTIP3.2 ---------------------------------------------------MATRRYSFG 09(60)

GmTIP3.3 ---------------------------------------------------MATRRYEFG 09(60)

GmTIP3.4 ---------------------------------------------------MATRRYEFG 09(60)

GmTIP4.1 -----------------------------------------------------MAKIALG 07(60)

GmTIP4.2 -----------------------------------------------------MARIALG 07(60)

GmNIP1.1 ---------------------------------MDENSATNGTHEVVLDVNRDVSRTTQA 27(60)

GmNIP1.2 ---------------------------------MDENSATNGTHEVILDVNKDVSRTTQP 27(60)

GmNIP2.1 ------------------------MEGTSSQSTFAFIPSTIETPSPSIPEISSSSSP--G 34(60)

GmNIP2.2 ------------------------MEGTTSQSTFTFIPSTIETPSPSIPEISSSSPSPGG 36(60)

GmNIP5.1 -----------------------MPESEIGTPTAASVPATPDTPGGPLFTSLRVDSLSHE 37(60)

GmNIP6.2 ---------------------------MDNNEEIPSTPATPGTPGAPLFGGFSNGRNNNS 33(60)

**TM1**

AtTIP1.1 RPDEATRP-------------------------------DALKAALAEFISTLIFVVAGS 38(120)

GmPIP1.1 EPGELSSW-------------------------------SFYRAGIAEFVATFLFLYITV 68(120)

GmPIP1.2 EPRELTSW-------------------------------SFYRAGIAEFVATFLFLYVTV 88(120)

GmPIP1.3 EPGELKSW-------------------------------SFYRAGIAEFVATFLFLYITV 68(120)

GmPIP1.4 EPGELKSW-------------------------------SFYRAGIAEFVATFLFLYITV 68(120)

GmPIP1.5 EPGELKSW-------------------------------SFYRAGIAEFVATFLFLYITI 69(120)

GmPIP1.6 EPGELKSW-------------------------------SFYRAGIAEFVATFLFLYITI 68(120)

GmPIP1.7 DPTEFTSW-------------------------------SFYRAGIAEFVATFLFLYITV 72(120)

GmPIP1.8 EPSELTSW-------------------------------SFYRAGIAEFVATFLFLYITI 72(120)

GmPIP2.1 DLAEIKLW-------------------------------SFYRALIAEFIATLLFLYVTV 50(120)

GmPIP2.2 DLAEIKLW-------------------------------SFYRALIAEFIASLLFLYVTV 53(120)

GmPIP2.7 DPAELRKW-------------------------------SFYRALIAEFVATLLFLYVTI 56(120)

GmPIP2.8 DPAELRKW-------------------------------SFFRALIAEFVATLLFLYVTI 56(120)

GmPIP2.9 DAEELTQW-------------------------------SFYRALIAEFIATLLFLYITV 54(120)

GmPIP2.10 DAEELTQW-------------------------------SFYRALIAEFIATMLFLYITV 54(120)

GmPIP2.11 DAEELTQW-------------------------------SFYRALIAEFIATMLFLYITV 54(120)

GmPIP2.12 DAEELTQW-------------------------------SFYRALIAEFIATLLFLYITV 54(120)

GmPIP2.13 DAEELTKW-------------------------------SFYRALIAEFIATLLFLYITV 65(120)

GmPIP2.14 DAEELTKW-------------------------------SFYRALIAEFIATLLFLYITV 56(120)

GmTIP1.1 TPGEAGQP-------------------------------DAIRAAFAEFFSMIIFVFAGE 38(120)

GmTIP1.2 TPGEAAQP-------------------------------DAIRAAFAEFFCMIIFVFAGE 38(120)

GmTIP1.4 NPSEFGQA-------------------------------DALKAALAEFISMLIFVFAGE 38(120)

GmTIP1.5 NPSEFGQA-------------------------------DALKAALAEFISMLIFVFAGE 38(120)

GmTIP1.6 NSSELNQS-------------------------------DALKAALAEFISMLIFVFAGE 38(120)

GmTIP1.7 RPEEATHP-------------------------------DTLKAGLAEFISTLIFVFAGS 38(120)

GmTIP1.8 RPEEATHP-------------------------------DTLKAALAEFISTFIFVFAGS 38(120)

GmTIP3.1 RADEATHP-------------------------------DSMRATLAEFVSTFIFVFAGE 38(120)

GmTIP3.2 RADEATHP-------------------------------DSMRATLAEFASTFIFVFAGE 38(120)

GmTIP3.3 RMNEASHP-------------------------------DSIRAALAEFLSTCIFVFAGE 38(120)

GmTIP3.4 RMNEASHP-------------------------------DSIRAALVEFLSTCIFVFAGE 38(120)

GmTIP4.1 STREATQP-------------------------------DCIQALIVEFIATFLFVFVGV 36(120)

GmTIP4.2 STREATQP-------------------------------DCIQALIVEFIATFLFVFVGV 36(120)

GmNIP1.1 SRSCVN--------------------------------VSFLQKLVAEVVGTYFLIFAGS 55(120)

GmNIP1.2 SRSCVN--------------------------------VSFLQKLVAEVVGTYFLIFAGC 55(120)

GmNIP2.1 SLARIAQS----------------------------YPPGFPRKVLAEIIGTFLLVFVGS 66(120)

GmNIP2.2 SLARVAQS----------------------------YPPGFPRKVFAEVIGTFLLVFVGS 68(120)

GmNIP5.1 RESFSMAG----------------CKCLPTKGHSCFTDFSMNKQVGAEFVGTFILIFAAT 81(120)

GmNIP6.2 KKSLLKSCRCFSVEEWSLEDGGLPAVSCSLPLPSPPPVVPLARKIGAEFIGTFILMFAGT 93(120)

: .* :::

**TM1**

**TM2**

**HB**

AtTIP1.1 GSGMAFNKLTENGA----TTPSGLVAAAVAHAFGLFVAVSVGANISGGHVNPAVTFGAFI 94(180)

GmPIP1.1 LTVMGVFKSK------SKCSTVGIQGIAWAFGGMIFALVYSTAGISGGHINPAVTFGLFL 122(180)

GmPIP1.2 LTVMGVAKSP------SKCSTVGVQGIAWSFGGMIFALVYCTAGISGGHINPAVTFGLFL 142(180)

GmPIP1.3 LTVMGVNRAP------NKCSSVGIQGIAWAFGGMIFALVYCTAGISGGHINPAVTFGLFL 122(180)

GmPIP1.4 LTVMGVNRAP------NKCSSVGIQGIAWAFGGMIFALVDCTAGISGGHINPAVTFGLFL 122(180)

GmPIP1.5 LTVMGVNRSP------SKCASVGIQGIAWAFGGMIFALVYCTAGISGGHINPAVTFGLFL 123(180)

GmPIP1.6 LTVMGVNRSP------SKCASVGIQGIAWAFGGMIFALVYCTAGISGGHINPAVTFGLFL 122(180)

GmPIP1.7 LTVMGVAGAK------SKCSTVGIQGIAWAFGGMIFALVYCTAGISGGHINPAVTFGLFL 126(180)

GmPIP1.8 LTVMGVNRSS------SKCATVGIQGIAWAFGGMIFALVYCTAGISGGHINPAVTFGLFL 126(180)

GmPIP2.1 ATVIGHKKQTGP------CDGVGLLGIAWAFGGMIFVLVYCTAGISGGHINPAVTFGLFL 104(180)

GmPIP2.2 ATIIGHKKQTGP------CDGVGLLGIAWSFGGMIFVLVYCTAGISGGHINPAVTFGLFL 107(180)

GmPIP2.7 LTVIGYNHQTATGS-PDLCNGVGVLGIAWAFGGMIFVLVYCTAGISGGHINPAVTFGLFL 115(180)

GmPIP2.8 LTVIGYNHQTAT-A-AEPCSGVGVLGIAWAFGGMIFVLVYCTAGISGGHINPAVTFGLFL 114(180)

GmPIP2.9 LTVIGYKSQSDVKAGGDVCGGVGILGIAWAFGGMIFILVYCTAGISGGHINPAVTFGLFL 114(180)

GmPIP2.10 LTVIGYKSQSDVKAGGDVCGGVGILGIAWAFGGMIFILVYCTAGISGGHINPAVTFGLFL 114(180)

GmPIP2.11 LTVIGYKSQSDVKAGGDVCGGVGILGIAWAFGGMIFILVYCTAGISGGHINPAVTFGLFL 114(180)

GmPIP2.12 LTVIGYKSQSDVKAGGDVCGGVGILGIAWAFGGMIFILVYCTAGISGGHINPAVTFGLFL 114(180)

GmPIP2.13 LTVIGYNHQTDLKENGEICGGVGILGIAWAFGGMIFILVYCTAGISGGHINPAVTFGLFL 125(180)

GmPIP2.14 LTVIGYKHQTDHAD---ACGGVGILGIAWAFGGMIFILVYCTAGISGGHINPAVTFGLFL 113(180)

GmTIP1.1 GSGMAYSKLTNNGP----ATPAGLIAASLSHAFGLFVAVSVGANISGGHVNPAVTFGAFI 94(180)

GmTIP1.2 GSGMAYSKLTNNGP----ATPAGLIAASLSHAFGLFVAVSVGANISGGHVNPAVTFGAFI 94(180)

GmTIP1.4 GSGMAYNKLTDNGS----ATPAGVVAASLSHAFALFVAVSVGANISGGHVNPAVTFGAFI 94(180)

GmTIP1.5 GSGMAYNKLTDNGS----ATPAGVVAASLSHAFALFVAVSVGANISGGHVNPAVTFGAFI 94(180)

GmTIP1.6 GSGMAYNKLTNNGS----ATPAGLVAASLSHAFALFVAVSVGANISGGHVNPAVTFGAFV 94(180)

GmTIP1.7 GSGIAYNKLTDNGA----ATPAGLISASIAHAFALFVAVSVGANISGGHVNPAVTFGAFV 94(180)

GmTIP1.8 GSGIAYNKLTDNGA----ATPAGLISASIAHAFALFVAVSVGANISGGHVNPAVTFGAFV 94(180)

GmTIP3.1 GSGLALVKIYQDSA----FSAGELLAVALAHGFALFAAVSASMHVSGGHVNPAVTFGALI 94(180)

GmTIP3.2 GSSLALVKIYQDSA----FSAGELLAVALAHAFALFAAVSSSMHVSGGHVNPAVTFGALI 94(180)

GmTIP3.3 GSALALRQIYKEPG----SSAGELVVIALAHAFALFAAISASMHVSGGHVNPAVTFGALL 94(180)

GmTIP3.4 GSALALRQIYKEPG----SSAGELVVIALAHAFALFAAISASMHVSGGHVNPAVTFGALL 94(180)

GmTIP4.1 GSSMVVDKLG---G----DALVGLFAVAVAHALVVAVMISA-AHISGGHLNPAVTLGLLA 89(180)

GmTIP4.2 ASSMVVDKLG---G----DALVGLFAVAVAHALVVAVMISA-AHISGGHLNPAVTLGLLA 89(180)

GmNIP1.1 ASVVVNKNN---------NNVVTLPGISIVWGLVVMVLVYSVGHISGAHFNPAVTIAFAS 106(180)

GmNIP1.2 ASVVVNKNN---------NNVVTHPGISIVWGLVVMVLVYSVGHISGAHFNPAVTIAFAS 106(180)

GmNIP2.1 GSAGLSKID---------ERMVSKLGASLAGGLIVTVMIYSIGHISGAHMNPAVSLAFTA 117(180)

GmNIP2.2 GSAGLSKID---------ESMVSKLGASLAGGLIVTVMIYSIGHISGAHMNPAVSLAFTA 119(180)

GmNIP5.1 AGPIVNNKY---------NGVESLMGNAACAGLTVMFIILSIGHISGAHLNPSLTIAFAA 132(180)

GmNIP6.2 AAAIVNQKT---------NGSETLIGCAATTGLAVMIVILATGHISGAHLNPAVTISFAA 144(180)

: . : : :**.*.**::::.

**TM3**

**TM4**

AtTIP1.1 GGNITLLRGILYWIAQLLGSVVACLILKFATGGLAVPAFG-----LSAGVGVLNAFVFEI 149(240)

GmPIP1.1 ARKLSLTRAIFYIIMQCLGAICGAGVVKGFE-PHLYERLGGGANTIAKGYTNSAGLGAEI 181(240)

GmPIP1.2 ARKLSLTRTVFYMIMQCLGAICGAAVVKGFQ-SNQYERLGGGANTLSKGYSKGDGLGAEI 201(240)

GmPIP1.3 ARKLSLTRAVFYIVMQCLGAICGAGVVKGFEGNARYELFKGGANFVSHGYTKGDGLGAEI 182(240)

GmPIP1.4 ARKLSLTRALFYIVMQCLGAICGAGVVKGFEGNARYELFKGGANFVSHGYTKGDGLGAEI 182(240)

GmPIP1.5 ARKLSLTRALFYIIMQCLGAICGAGVVKGFEGNARYEMFKGGANFVNSGYTKGDGLGAEI 183(240)

GmPIP1.6 ARKLSLTRALFYIIMQCLGAICGAGVVKGFEGNANYELFKGGANFVNSGYTKGDGLGAEI 182(240)

GmPIP1.7 ARKLSLPRAIFYIVMQCLGAICGAGVVKGFEGKTKYGALNGGANFVAPGYTKGDGLGAEI 186(240)

GmPIP1.8 ARKLSLTRALFYMVMQVLGAIVGAGVVKGFEGKTFYGQHNGGANFVAPGYTKGDGLGAEI 186(240)

GmPIP2.1 ARKVSLIRALFYMVAQCLGAICGVGLVKAFMK-HSYNSLGGGANSVSAGYNKGSALGAEI 163(240)

GmPIP2.2 ARKVSLIRAVFYMVAQCLGAICGVGLVKAFMK-HSYNSLGGGANSVSAGYNKGSALGAEI 166(240)

GmPIP2.7 ARKVSLIRAVGYMVAQVLGAISGVGLVKALQK-SYYNRYNGGVNMLADGYSKGTGLGAEI 174(240)

GmPIP2.8 ARKVSLTRAVGYMVAQVLGAISGVGLVKALQK-SYYNRYKGGVNMLADGYSKGTGLGAEI 173(240)

GmPIP2.9 ARKVSLIRAIMYMVAQCLGAICGVGLVKAFQK-AYYNRYGGGANELSEGYSTGVGLGAEI 173(240)

GmPIP2.10 ARKVSLIRAIMYMVAQCLGAICGVGLVKAFQK-AYYNRYGGGANELSEGYSTGVGLGAEI 173(240)

GmPIP2.11 ARKVSLIRAIMYMVAQCLGAICGVGLVKAFQK-AYYNRYGGGANELSEGYSTGVGLGAEI 173(240)

GmPIP2.12 ARKVSLIRAIMYMVAQCLGAMCGVGLVKAFQK-AYYNRYGGGANELSEGYSTGVGLGAEI 173(240)

GmPIP2.13 ARKVSLIRAIMYMVAQCLGAICGVGLVKAFQK-SYFNKYGGGANSLAAGYSTGTGLGAEI 184(240)

GmPIP2.14 ARKVSLIRAIMYMVAQCLGAICGVGLVKAFQK-SYFNKYGGGANSLADGYSTGTGLGAEI 172(240)

GmTIP1.1 GGNITLLRSILYWIAQLLGSVVACILLKSATGGMETTGFS-----LSPGVSVWNALVFEI 149(240)

GmTIP1.2 GGNITLLRSILYWIAQLFGSVVACILLKHATGGMETSGFS-----LSPGVSVWNALVFEI 149(240)

GmTIP1.4 GGHISLLRGILYWIAQLLGSVVACLLLKFATGGLETSAFS-----LSPGVGAANALVFEI 149(240)

GmTIP1.5 GGHISLLRGILFWIAQLLGSVVACLLLKFAT-------VG-----LSPGVGAANALVFEI 142(240)

GmTIP1.6 GGHITLFRSILYWIAQLLGSVVACLLLKFATGGLETSAFA-----LSPGVEAGNALVFEI 149(240)

GmTIP1.7 GGNITLLRGIVYVIAQLLGSIVASLLLAFVT-ASPVPAFG-----LSAGVGVGNALVLEI 148(240)

GmTIP1.8 GGNITFLRGIVYVIAQLLGSIVASLLLAFVT-ASTVPAFG-----LSAGVGVGNALVLEI 148(240)

GmTIP3.1 GGRISVLRAVYYWIAQILGAIVAALVLRLVTNNMRPSGFH-----VGQGVGVGHMLILEI 149(240)

GmTIP3.2 GGRISVLRAVYYWIAQILGAIVAALVLRLVTNNMRPSGFH-----VGQGVGVGHMLILEI 149(240)

GmTIP3.3 GGRISVLRAVYYWVAQLLGSIVAALLLRLVTNNMRPQGFS-----VSIGLGAFHGLVLEI 149(240)

GmTIP3.4 GGRISVLRALYYWVAQLLGSIVAALLLRLVTNNMRPQGFS-----VSIGLGAFHGLILEI 149(240)

GmTIP4.1 GGHITIFRSMLYWIDQLVAAATASYLLYYLSGGQATPVHT-----LASGVGYGQGVVWEI 144(240)

GmTIP4.2 GGHITIFRSLLYWIDQLVAAAAASYLLYYLSGGQATPVHT-----LASGVGYGQGVVWEI 144(240)

GmNIP1.1 TKRFPLKQVPVYVVAQVVGSTLASGTLRLLFSGKEAQFSG-----TLPSGSNLQAFVIEF 161(240)

GmNIP1.2 TRRFPLKQVPVYVVAQVVGSTLASATLRLLFSGKETQFSG-----TLPSGSNLQAFVIEF 161(240)

GmNIP2.1 VRHLPWPQLPFYIAAQLTGAISASYTLRELLRPSNEIG-G-----TSPAGSHIQALIMEM 171(240)

GmNIP2.2 VRHLPWPQLPFYVAAQLTGAISASYTLRELLRPSDEIG-G-----TSPAGSHIQALIMEM 173(240)

GmNIP5.1 FRHFPWTHVPAYIAAQVSASICACYALKGVYHPFLSGG-V-----TVPTVSVAQAFATEF 186(240)

GmNIP6.2 LKHFPWKHVPMYIGAQVLASICAGFALKGVYHPFMSGG-V-----TVPSGGYGQSFALEF 198(240)

... : : * .: . : . *:

**HE**

**TM4**

**TM5**

AtTIP1.1 VMTFGLVYTVYATAIDPKNGS----LGTIAPIAIGFIVGANILAGGAFSGASMNPAVAFG 205(300)

GmPIP1.1 VGTFVLVYTVFS-ATDAKRNARDSHVPILAPLPIGFAVFLVHLATIPVTGTGINPARSLG 240(300)

GmPIP1.2 VGTFILVYTVFS-ATDAKRNARDSHVPILAPLPIGFAVFLVHLATIPITGTGINPARSLG 260(300)

GmPIP1.3 VGTFILVYTVFS-ATDAKRNARDSHVPILAPLPIGFAVFLVHLATIPITGTGINPARSLG 241(300)

GmPIP1.4 VGTFILVYTVFS-ATDAKRNARDSHVPILAPLPIGFAVFLVHLATIPITGTGINPARSLG 241(300)

GmPIP1.5 VGTFVLVYTVFS-ATDAKRNARDSHVPILAPLPIGFAVFLVHLATIPITGTGINPARSLG 242(300)

GmPIP1.6 VGTFVLVYTVFS-ATDAKRNARDSHVPILAPLPIGFAVFLVHLATIPITGTGINPARSLG 241(300)

GmPIP1.7 VGTFILVYTVFS-ATDAKRSARDSHVPILAPLPIGFAVFLVHLATIPITGTGINPARSLG 245(300)

GmPIP1.8 VGTFILVYTVFS-ATDAKRSARDSHVPILAPLPIGFAVFLVHLATIPITGTGINPARSLG 245(300)

GmPIP2.1 IGTFVLVYTVFS-ATDPKRSARDSHIPVLAPLPIGFAVFMVHLATIPITGTGINPARSFG 222(300)

GmPIP2.2 IGTFVLVYTVFS-ATDPKRSARDSHVPVLAPLPIGFAVFMVHLATIPITGTGINPARSLG 225(300)

GmPIP2.7 IGTFILVYTVFS-ATDPKRVARDSHVPVLAPLPIGFAVFIVHLATIPITGTGINPARSLG 233(300)

GmPIP2.8 IGTFILVYTVFS-ATDPKRVARDSHVPVLAPLPIGFAVFMVHLATIPITGTGINPARSLG 232(300)

GmPIP2.9 IGTFVLVYTVFS-ATDPKRNARDSHVPVLAPLPIGFAVFMVHLATIPVTGTGINPARSLG 232(300)

GmPIP2.10 IGTFVLVYTVFS-ATDPKRNARDSHVPVLAPLPIGFAVFMVHLATIPVTGTGINPARSLG 232(300)

GmPIP2.11 IGTFVLVYTVFS-ATDPKRNARDSHVPVLAPLPIGFAVFMVHLATIPVTGTGINPARSLG 232(300)

GmPIP2.12 IGTFVLVYTVFS-ATDPKRNARDSHVPVLAPLPIGFAVFMVHLATIPVTGTGINPARSFG 232(300)

GmPIP2.13 IGTFVLVYTVFS-ATDPKRNARDSHVPVLAPLPIGFAVFMVHLATIPVTGTGINPARSLG 243(300)

GmPIP2.14 IGTFVLVYTVFS-ATDPKRNARDSHVPVLAPLPIGFAVFMVHLATIPVTGTGINPARSLG 231(300)

GmTIP1.1 VMTFGLVYTVYATAVDPKKGN----VGVVAPIAIGFIVGANILVGGAFDGASMNPAVSFG 205(300)

GmTIP1.2 VMTFGLVYTVYATAVDPKKGN----AGVVAPIAIGFIVGANILVGGAFDGASMNPAVSFG 205(300)

GmTIP1.4 VMTFGLVYTVYATAVDPKKGN----LGIIAPIAIGFIVGANILAGGAFDGASMNPAVSFG 205(300)

GmTIP1.5 VMTFGLVYTVYATAVDPKKGK----LGIIAPIAIGFIVGANILAGGTFSGASMNPAVSFG 198(300)

GmTIP1.6 VMTFGLVYTVYATAVDPKKGD----LGIIAPIAIGFIVGANILAGGAFDGASMNPAVSFG 205(300)

GmTIP1.7 VMTFGLVYTVYATAVDPKKGN----LGIIAPIAIGFIVGANILLGGAFSGAAMNPAVTFG 204(300)

GmTIP1.8 VMTFGLVYTVYATAIDPKKGN----LGIIAPIAIGFIVGANILLGGAFSGAAMNPAVTFG 204(300)

GmTIP3.1 VMTFGLMYTVYGTAIDPKRGA----VSNIAPLAIGLIVGANILVGGPFDGACMNPALAFG 205(300)

GmTIP3.2 IMTFGLMYTVYGTAIDPKRGS----VSNIAPLAIGLIVGANILVGGPFDGACMNPALAFG 205(300)

GmTIP3.3 ALTFGLMYTVYATAIDPKRGS----IGSIAPLAIGFVVGANILAGGPFDGACMNPARAFG 205(300)

GmTIP3.4 ALTFGLMYTVYATAIDPKRGS----IGSIAPLAIAFVVGANILAGGPFDGACMNPARAFG 205(300)

GmTIP4.1 VLTFSLLFTVYATMVDPKKGA----LAGLGPTLVGFVVGANILAGGAYSAASMNPARSFG 200(300)

GmTIP4.2 VLTFSLLFTVYATMVDPKKGA----LAGLGPTLVGFVVGANILAGGAYSAASMNPARSFG 200(300)

GmNIP1.1 LITFFLMFVVSGVATDNR------AIGELAGIAVGSTVLLNVMFAGPITGASMNPARSIG 215(300)

GmNIP1.2 LITFFLMFVISGVATDDR------AIGELAGIAVGSTVLLNVMFAGPITGASMNPARSIG 215(300)

GmNIP2.1 VTTYTMVFISMAVATDSN------ATGQLSGVAVGSSVCIASIVAGPISGGSMNPARTLG 225(300)

GmNIP2.2 VSTYTMVFISMAVATDSN------ATGQLSGVAVGSSVCIASIVAGPISGGSMNPARTLG 227(300)

GmNIP5.1 IITFILLFVVTAVATDTR------AVGELAGIAVGATVLLNILISGPTSGGSMNPVRTLG 240(300)

GmNIP6.2 IIGFNLMFVVTAVATDTR------AVGELAGIAVGATVMLNILIAGPVSGGSMNPVRTLG 252(300)

: ::: . * . :. :. : : . . :**. ::*

**TM6**

AtTIP1.1 PAVVSWT---WTNHWVYWAGPLVGGGIAGLIYEVFFIN----TTHEQLPTTDY------- 251(360)

GmPIP1.1 AAIIFNKDQAWDDHWIFWVGPFIGAALAALYHQIVIRAIPFSSK---------------- 284(360)

GmPIP1.2 AALVYNKDQAWDNHWIFWVGPFIGAALAALYHQIVLRAIPFKSK---------------- 304(360)

GmPIP1.3 AAIIYNRDHAWDDHWIFWVGPFIGAALAAVYHQIVIRAIPFKTRG--------------- 286(360)

GmPIP1.4 AAIIYNRDHAWDDHWIFWVGPFIGAALAALYHQIVIRAIPFKTRG--------------- 286(360)

GmPIP1.5 AAIIYNRDHAWDDQWIFWVGPFIGAALAAVYHQIVIRAIPFKTRA--------------- 287(360)

GmPIP1.6 AAIIYNRDHAWDDQWIFWVGPFIGAALAAVYHQIVIRAIPFKTRA--------------- 286(360)

GmPIP1.7 AAIIFNKDLGWDEHWIFWVGPFIGAALAALYHQVVIRAIPFKSK---------------- 289(360)

GmPIP1.8 AAIIFNKDLGWDDHWIFWVGPFVGAALAALYHQVVIRAIPFKSS---------------- 289(360)

GmPIP2.1 AAVIYNNGKVWDDHWIFWVGPFVGALAAAAYHQYILRAAAIKALGSFRSNPTN------- 275(360)

GmPIP2.2 AAVIYNNGKVWDEHWIFWVGPLVGALAAAAYHQYILRAGAIKALGSFRSNPTN------- 278(360)

GmPIP2.7 PAVIFNNEKAWDDQWIFWVGPFIGAAIAAFYHQSVLRAQAAKALGSFRSSSNL------- 286(360)

GmPIP2.8 PAVIFNNEKAWDDQWIFWVGPFIGAALAAFYHQSVLRAQAAKALGSFRSSSNL------- 285(360)

GmPIP2.9 AAVMYNQQKAWDDHWIFWVGPFIGAAIAAFYHQFILRAGAAKALGSFRSNPTI------- 285(360)

GmPIP2.10 AAVMYNQQKAWDDHWIFWVGPFIGAAIAAFYHQFILRAGAAKALGSFRSNPAI------- 285(360)

GmPIP2.11 AAVMYNQQKAWDDHWIFWVGPFIGAAIAAFYHQFILRASAAKALGSFRSNPTI------- 285(360)

GmPIP2.12 AAVMYNQKKAWDDQWIFWVGPFIGAAIAAFYHQFILRASAAKAVGSFRSNPTI------- 285(360)

GmPIP2.13 AAVIYNQDKPWDDHWIFWVGPFIGAAIAAFYHQFILRAGAAKALGSFRSNPHN------- 296(360)

GmPIP2.14 AAVIYNQDKPWDDHWIFWVGPFIGAAIAAFYHQFILRAGAAKALGSFRSNPHN------- 284(360)

GmTIP1.1 PAVVTWS---WTHHWVYWVGPFIGAAIAAVIYDNIFIG---DDGHEPLSSSDF------- 252(360)

GmTIP1.2 PAVVTWS---WTHHWVYWVGPFIGAAIAAIIYDNIFIG---DDGHEPLSSSDF------- 252(360)

GmTIP1.4 PAVVSGT---WANHWVYWVGPLIGSAIAAIIYETFFIT---PNSYEHLPVTDY------- 252(360)

GmTIP1.5 PAVVSGT---WANHWVYWAGPLIGSAIAAVVYETFFIT---PNSYEQLPVTDY------- 245(360)

GmTIP1.6 PAVVSWT---WSNHWVYWVGPFAGAAIAAVVYEIFFIS---PNTHEQLPVTDY------- 252(360)

GmTIP1.7 PAVVSWT---WTNHWIYWAGPLIGGGIAGLIYEVVFIS----HTHEQLPSTDY------- 252(360)

GmTIP1.8 PAVVSWT---WTNHWIYWAGPLIGGGIAGLVYEVVFIS----HTHEQLPTTDY------- 252(360)

GmTIP3.1 PSLVGWR---WHQHWIFWVGPLIGAALAALVYEYVVIPTEPPHQHQPLAPEDY------- 255(360)

GmTIP3.2 PSLVGWR---WHQHWIFWVGPLIGAALAALVYEYVVIPTEPPHQHQPLAPEDY------- 255(360)

GmTIP3.3 PAMVGWR---WHYHWIFWVGPFIGAALAALLYEYVMVPNEPP-HHQPLAAEDY------- 254(360)

GmTIP3.4 PAMVGWR---WHYHWIFWVGPLIGAALAALLYEYVMVPIEPP-HHQPLAGVDY------- 254(360)

GmTIP4.1 PALVAGN---WTDHWVYWVGPLIGGGLAGYIYETFFIDR----SHVPLPRDEEN------ 247(360)

GmTIP4.2 PALVTGN---WTDHWVYWVGPLIGGGLAGFIYETFFIDR----SHVPLPRDEES------ 247(360)

GmNIP1.1 PAIVHK---EYRGIWIYLVSPTLGAVAGAWVYNSIRYT--DKPLREITKSASFLKGVASR 270(360)

GmNIP1.2 PAILHN---EYRGIWIYIVSPTLGAVAGTWVYNTIRYT--DKPLREITKSTSFLKGVGRS 270(360)

GmNIP2.1 PAIATS---YYKGLWVYFVGPITGAVLAAWSYNVIRDTEHPGFPISLSSISSKVRQSIGG 282(360)

GmNIP2.2 PAIATS---YYKGLWVYFVGPITGAVLAAWSYNVIRDTEHPGFPISLSSISSKVRQSIGG 284(360)

GmNIP5.1 PAVAAG---NYKHIWIYLVAPTLGALAGAGVYTLVKLR--DEEAEPPRQVRSFRR----- 290(360)

GmNIP6.2 PAVAAN---NYKAIWVYLVAPILGALAGAGTYTAVKLPEEDDDAKAKTSISSFRRCNTEN 309(360)

.:. : *:: .* *. . : .

AtTIP1.1 ------------------------------------------------------------ 251(420)

GmPIP1.1 ------------------------------------------------------------ 284(420)

GmPIP1.2 ------------------------------------------------------------ 304(420)

GmPIP1.3 ------------------------------------------------------------ 286(420)

GmPIP1.4 ------------------------------------------------------------ 286(420)

GmPIP1.5 ------------------------------------------------------------ 287(420)

GmPIP1.6 ------------------------------------------------------------ 286(420)

GmPIP1.7 ------------------------------------------------------------ 289(420)

GmPIP1.8 ------------------------------------------------------------ 289(420)

GmPIP2.1 ------------------------------------------------------------ 275(420)

GmPIP2.2 ------------------------------------------------------------ 278(420)

GmPIP2.7 ------------------------------------------------------------ 286(420)

GmPIP2.8 ------------------------------------------------------------ 285(420)

GmPIP2.9 ------------------------------------------------------------ 285(420)

GmPIP2.10 ------------------------------------------------------------ 285(420)

GmPIP2.11 ------------------------------------------------------------ 285(420)

GmPIP2.12 ------------------------------------------------------------ 285(420)

GmPIP2.13 ------------------------------------------------------------ 296(420)

GmPIP2.14 ------------------------------------------------------------ 284(420)

GmTIP1.1 ------------------------------------------------------------ 252(420)

GmTIP1.2 ------------------------------------------------------------ 252(420)

GmTIP1.4 ------------------------------------------------------------ 252(420)

GmTIP1.5 ------------------------------------------------------------ 245(420)

GmTIP1.6 ------------------------------------------------------------ 252(420)

GmTIP1.7 ------------------------------------------------------------ 252(420)

GmTIP1.8 ------------------------------------------------------------ 252(420)

GmTIP3.1 ------------------------------------------------------------ 255(420)

GmTIP3.2 ------------------------------------------------------------ 255(420)

GmTIP3.3 ------------------------------------------------------------ 254(420)

GmTIP3.4 ------------------------------------------------------------ 254(420)

GmTIP4.1 ------------------------------------------------------------ 247(420)

GmTIP4.2 ------------------------------------------------------------ 247(420)

GmNIP1.1 ------------------------------------------------------------ 270(420)

GmNIP1.2 GSSR-------------------------------------------------------- 274(420)

GmNIP2.1 TEQKSDQRCLV------------------------------------------------- 292(420)

GmNIP2.2 TEQKSDQRCLV------------------------------------------------- 294(420)

GmNIP5.1 ------------------------------------------------------------ 290(420)

GmNIP6.2 T-DISPSITINFRM-DATANAMESEQFILEFILENAMECNTENT-TYPEAPPLICATINC 366(420)

AtTIP1.1 ---------- 251(480)

GmPIP1.1 ---------- 284(480)

GmPIP1.2 ---------- 304(480)

GmPIP1.3 ---------- 286(480)

GmPIP1.4 ---------- 286(480)

GmPIP1.5 ---------- 287(480)

GmPIP1.6 ---------- 286(480)

GmPIP1.7 ---------- 289(480)

GmPIP1.8 ---------- 289(480)

GmPIP2.1 ---------- 275(480)

GmPIP2.2 ---------- 278(480)

GmPIP2.7 ---------- 286(480)

GmPIP2.8 ---------- 285(480)

GmPIP2.9 ---------- 285(480)

GmPIP2.10 ---------- 285(480)

GmPIP2.11 ---------- 285(480)

GmPIP2.12 ---------- 285(480)

GmPIP2.13 ---------- 296(480)

GmPIP2.14 ---------- 284(480)

GmTIP1.1 ---------- 252(480)

GmTIP1.2 ---------- 252(480)

GmTIP1.4 ---------- 252(480)

GmTIP1.5 ---------- 245(480)

GmTIP1.6 ---------- 252(480)

GmTIP1.7 ---------- 252(480)

GmTIP1.8 ---------- 252(480)

GmTIP3.1 ---------- 255(480)

GmTIP3.2 ---------- 255(480)

GmTIP3.3 ---------- 254(480)

GmTIP3.4 ---------- 254(480)

GmTIP4.1 ---------- 247(480)

GmTIP4.2 ---------- 247(480)

GmNIP1.1 ---------- 270(480)

GmNIP1.2 ---------- 274(480)

GmNIP2.1 ---------- 292(480)

GmNIP2.2 ---------- 294(480)

GmNIP5.1 ---------- 290(480)

GmNIP6.2 TET-STREAM 375(480)

**Urea**

AtTIP1.1 ---------------------------------------------------MPIRNIAIG 09(60)

GmTIP1.4 ---------------------------------------------------MPISRIAIG 09(60)

GmTIP1.6 ---------------------------------------------------MPISRIAIG 09(60)

GmTIP1.1 ---------------------------------------------------MAIYRIAIG 09(60)

GmTIP1.2 ---------------------------------------------------MAMHRIAIG 09(60)

GmTIP1.3 ---------------------------------------------------MAVYRIAIG 09(60)

GmTIP1.9 ---------------------------------------------------MAYRSAIVR 09(60)

GmTIP2.1 -----------------------------------------------------MVKIALG 07(60)

GmTIP2.2 -----------------------------------------------------MVKITLG 07(60)

GmTIP2.3 -----------------------------------------------------MGGIAFG 07(60)

GmTIP2.4 -----------------------------------------------------MGGIAFG 07(60)

GmTIP2.5 -----------------------------------------------------MAGIAFG 07(60)

GmTIP2.6 -----------------------------------------------------MAGIAFG 07(60)

GmTIP2.7 -----------------------------------------------------MAGIAFG 07(60)

GmTIP5.1 --------------------------------------------------MAPSSVTVTS 10(60)

GmPIP2.10 -----------------------------------MAKHDVEGGSFSAKDYHDPPPAPLI 25(60)

GmPIP2.11 -----------------------------------MAKHDVEGGSFAAKDYHDPPPAPLI 25(60)

GmPIP2.9 -----------------------------------MAKHDVEGGSFSAKDYHDPPPAPLI 25(60)

GmPIP2.12 -----------------------------------MAKHDVEGGSFSAKDYHDPPPAPLI 25(60)

GmPIP2.13 ------------------------MSVFWQEGGMAKDVEVAERGSFSGKDYQDPPPAPLI 36(60)

GmPIP2.14 ---------------------------------MAKDVEVAERGSFSGKDYQDPPPAPLI 27(60)

GmPIP2.3 --------------------------------MAKDVEVQEQGGEYSAKDYHDPPPAPLF 28(60)

GmPIP2.4 --------------------------------MAKDVEVQEQGGEYSAKDYHDPPPAPLF 28(60)

GmPIP2.5 --------------------------------MAKDVEQVTEQGEYSAKDYHDPPPAPLI 28(60)

GmPIP2.6 --------------------------------MAKDVEQVTEQGEYSAKDYHDPPPAPLI 28(60)

GmPIP2.1 ------------------------------------MSKEVSQ---QRKDYVDPPPAPLI 21(60)

GmPIP2.2 ------------------------------------MSKEVSQEGLQRKDYVDPPPAPLF 24(60)

GmPIP2.7 ---------------------------------MAKDIETEVQSGLPHKDYHDPPAAAFY 27(60)

GmPIP2.8 ---------------------------------MAKDLETEIQSGLPHKDYHDPPPAPFY 27(60)

GmPIP1.1 -----------------MEGKEEDVRVGANRYGERQPIGTAAQ----AKDYREPPSAPLF 39(60)

GmPIP1.2 LRLPHSHIHIEVKLALKMEGRDEDVRVGANRYGERQPIGTAAQTQD-AKDYREAPPAPLF 59(60)

GmPIP1.3 ------------------MEREEDVKVGAQKFSERQALGTGAKS---DKDYKEAPPAPLF 39(60)

GmPIP1.4 ------------------MEREEDVKVGAQKFSERQALGTGAQG---DKDYKEAPPAPLF 39(60)

GmPIP1.5 -----------------MESKEEDVRVGATKFSERQPIGTAAQG---DKDYKEPPPAPLF 40(60)

GmPIP1.6 -----------------MESKEEDVNVGANKFSERQPIGTAAQGGG-DKDYKEAPPAPLF 42(60)

GmPIP1.7 -----------------MEGKEQDVSLGANKFPERQPIGTAAQSQDDGKDYQEPAPAPLV 43(60)

GmPIP1.8 -----------------MEGKEEDVSLGANKFSERQPIGTAAQSQDDGKDYTEPPPAPLF 43(60)

GmNIP1.1 -----------------------------------MDENSATNGTHEVVLDVNRDVSRTT 25(60)

GmNIP1.2 -----------------------------------MDENSATNGTHEVILDVNKDVSRTT 25(60)

GmNIP1.3 --------------------------------MSVVADNSANNGSHQVVLNVNGDAPKKC 28(60)

GmNIP1.4 --------------------------------------MYTNNGSHQVVLNVNGDASKKC 22(60)

GmNIP1.5Nod26 -----------------------------------MADYSAGTESQEVVVNVTKNTSETI 25(60)

GmNIP4.1 ------------------------------------------------MEENGGNIHADS 12(60)

GmNIP6.1 ---------------------------MNNEEVPSLPSTSATPGTPGAPLFGGLRFEKPN 33(60)

GmNIP6.2 -----------------------------MDNNEEIPSTPATPGTPGAPLFGGFSNGRNN 31(60)

GmNIP5.1 -------------------------MPESEIGTPTAASVPATPDTPGGPLFTSLRVDSLS 35(60)

**TM1**

AtTIP1.1 RPDEATR------------------------------------PDALKAALAEFISTLIF 33(120)

GmTIP1.4 NPSEFGQ------------------------------------ADALKAALAEFISMLIF 33(120)

GmTIP1.6 NSSELNQ------------------------------------SDALKAALAEFISMLIF 33(120)

GmTIP1.1 TPGEAGQ------------------------------------PDAIRAAFAEFFSMIIF 33(120)

GmTIP1.2 TPGEAAQ------------------------------------PDAIRAAFAEFFCMIIF 33(120)

GmTIP1.3 SPREASN------------------------------------PAAIRAAFAEFFSMLIF 33(120)

GmTIP1.9 RAQEASH------------------------------------RDTWRAALSEFISTLIF 33(120)

GmTIP2.1 TLDDSFS------------------------------------AASLKAYFAEFHATLIF 31(120)

GmTIP2.2 TFDDSFG------------------------------------VASLKAYLAEFHATLIF 31(120)

GmTIP2.3 RLDDSFS------------------------------------LTSIKAYIAEFHSTLLF 31(120)

GmTIP2.4 RFDDSFS------------------------------------LTSIKAYIAEFHSTLLF 31(120)

GmTIP2.5 SFNDSVS------------------------------------FASIKAYIAEFISTLLF 31(120)

GmTIP2.6 SFNDSFS------------------------------------LASIKAYIAEFISTLLF 31(120)

GmTIP2.7 NFNDSVS------------------------------------FASIKAYIAEFISTLLF 31(120)

GmTIP5.1 RFHESVT------------------------------------RNALRSYLSEFISTFFY 34(120)

GmPIP2.10 DAEELTQ------------------------------------WSFYRALIAEFIATMLF 49(120)

GmPIP2.11 DAEELTQ------------------------------------WSFYRALIAEFIATMLF 49(120)

GmPIP2.9 DAEELTQ------------------------------------WSFYRALIAEFIATLLF 49(120)

GmPIP2.12 DAEELTQ------------------------------------WSFYRALIAEFIATLLF 49(120)

GmPIP2.13 DAEELTK------------------------------------WSFYRALIAEFIATLLF 60(120)

GmPIP2.14 DAEELTK------------------------------------WSFYRALIAEFIATLLF 51(120)

GmPIP2.3 DPEELTQ------------------------------------WSFYRALIAEFIATLLF 52(120)

GmPIP2.4 DPEELTQ------------------------------------WSFYRALIAEFIATLLF 52(120)

GmPIP2.5 DPDELTK------------------------------------WSLYRAAIAEFIATLLF 52(120)

GmPIP2.6 DPDELTK------------------------------------WSLYRAAIAEFIATLLF 52(120)

GmPIP2.1 DLAEIKL------------------------------------WSFYRALIAEFIATLLF 45(120)

GmPIP2.2 DLAEIKL------------------------------------WSFYRALIAEFIASLLF 48(120)

GmPIP2.7 DPAELRK------------------------------------WSFYRALIAEFVATLLF 51(120)

GmPIP2.8 DPAELRK------------------------------------WSFFRALIAEFVATLLF 51(120)

GmPIP1.1 EPGELSS------------------------------------WSFYRAGIAEFVATFLF 63(120)

GmPIP1.2 EPRELTS------------------------------------WSFYRAGIAEFVATFLF 83(120)

GmPIP1.3 EPGELKS------------------------------------WSFYRAGIAEFVATFLF 63(120)

GmPIP1.4 EPGELKS------------------------------------WSFYRAGIAEFVATFLF 63(120)

GmPIP1.5 EPGELKS------------------------------------WSFYRAGIAEFVATFLF 64(120)

GmPIP1.6 EPGELKS------------------------------------WSFYRAGIAEFVATFLF 66(120)

GmPIP1.7 DPTEFTS------------------------------------WSFYRAGIAEFVATFLF 67(120)

GmPIP1.8 EPSELTS------------------------------------WSFYRAGIAEFVATFLF 67(120)

GmNIP1.1 QASRSCV-----------------------------------NVSFLQKLVAEVVGTYFL 50(120)

GmNIP1.2 QPSRSCV-----------------------------------NVSFLQKLVAEVVGTYFL 50(120)

GmNIP1.3 DDSANQD-----------------------------------CVPLLQKLVAEVVGTYFL 53(120)

GmNIP1.4 DDSSNQD-----------------------------------CVPLLQKLVAEVVGTYFL 47(120)

GmNIP1.5Nod26 QRSDSLV-----------------------------------SVPFLQKLVAEAVGTYFL 50(120)

GmNIP4.1 TFCGSPA-----------------------------------VVQVIQKVIAELIGTYFL 37(120)

GmNIP6.1 GSVVRKSSFLKSCKCFSVAEWTLEDGAMPRVSCSLPSP----HIPLAKKIGAEFIGTFIL 89(120)

GmNIP6.2 N---SKKSLLKSCRCFSVEEWSLEDGGLPAVSCSLPLPSPPPVVPLARKIGAEFIGTFIL 88(120)

GmNIP5.1 HE--RESFSMAGCKCLPTKGHSCFT-----------------DFSMNKQVGAEFVGTFIL 54(120)

: :* :

**TM1**

**TM2**

**HB**

AtTIP1.1 VVAGSGSGMAFNKLTE-----NGATTPSGLVAAAVAHAFGLFVAVSVGANISGGHVNPAV 88(180)

GmTIP1.4 VFAGEGSGMAYNKLTD-----NGSATPAGVVAASLSHAFALFVAVSVGANISGGHVNPAV 88(180)

GmTIP1.6 VFAGEGSGMAYNKLTN-----NGSATPAGLVAASLSHAFALFVAVSVGANISGGHVNPAV 88(180)

GmTIP1.1 VFAGEGSGMAYSKLTN-----NGPATPAGLIAASLSHAFGLFVAVSVGANISGGHVNPAV 88(180)

GmTIP1.2 VFAGEGSGMAYSKLTN-----NGPATPAGLIAASLSHAFGLFVAVSVGANISGGHVNPAV 88(180)

GmTIP1.3 VFAGQGSGMAYSKLTG-----NGPATPGGLVVASLSHTFGLFVAVAVGANISGGHVNPAV 88(180)

GmTIP1.9 VFAGSGSSVAVNKLTV-----D---KPSALVVAAVAHAFALFVAVSVSTNISGGHVNPAV 85(180)

GmTIP2.1 VFAGVGSAIAYNELTK-----DAALDPTGLVAVAVAHAFALFVGVSVAANISGGHLNPAV 86(180)

GmTIP2.2 VFAGVGSAIAYNELTK-----DAALDPTGLVAVAVAHAFALFVGVSVAANISGGHLNPAV 86(180)

GmTIP2.3 VFAGVGSAIAYGKLTS-----DAALDPAGLLAVAICHGFALFVAVSVGANISGGHVNPAV 86(180)

GmTIP2.4 VFAGVGSAIAYGKLTS-----DAALDPAGLLAVAICHGFALFVAVSVGANISGGHVNPAV 86(180)

GmTIP2.5 VFAGVGSAIAYAKLTS-----DAALDPTGLVAVAICHGFALFVAVSVGANISGGHVNPAV 86(180)

GmTIP2.6 VFAGVGSAIAYAKLTS-----DAALDPTGLVAVAICHGFALFVAVSVGANISGGHVNPAV 86(180)

GmTIP2.7 VFAGVGSAIAYAKLTS-----DAALDPTGLVAVAICHGFALFVAVSVGANISGGHVNPAV 86(180)

GmTIP5.1 VFLVIGAGMSSRKLMP-----DASLNPTSLVVVGIGSAFALSSVLYIAWDISGGHVNPAV 89(180)

GmPIP2.10 LYITVLTVIGYKSQSDVKA-GGDVCGGVGILGIAWAFGGMIFILVYCTAGISGGHINPAV 108(180)

GmPIP2.11 LYITVLTVIGYKSQSDVKA-GGDVCGGVGILGIAWAFGGMIFILVYCTAGISGGHINPAV 108(180)

GmPIP2.9 LYITVLTVIGYKSQSDVKA-GGDVCGGVGILGIAWAFGGMIFILVYCTAGISGGHINPAV 108(180)

GmPIP2.12 LYITVLTVIGYKSQSDVKA-GGDVCGGVGILGIAWAFGGMIFILVYCTAGISGGHINPAV 108(180)

GmPIP2.13 LYITVLTVIGYNHQTDLKE-NGEICGGVGILGIAWAFGGMIFILVYCTAGISGGHINPAV 119(180)

GmPIP2.14 LYITVLTVIGYKHQTDHAD----ACGGVGILGIAWAFGGMIFILVYCTAGISGGHINPAV 105(180)

GmPIP2.3 LYVTVLTIIGYKRQTDATLGG-TECDGVGILGIAWAFGGMIFILVYCTAGISGGHINPAV 111(180)

GmPIP2.4 LYVTVLTIIGYKRQTDTTVGG-TDCDGVGILGIAWAFGGMIFILVYCTAGISGGHINPAV 111(180)

GmPIP2.5 LYITVLTIIGYKRQSDTKIPGNTECDGVGILGIAWAFGGMIFILVYCTAGISGGHINPAV 111(180)

GmPIP2.6 LYITVLTIIGYKRQSDTKIPGNTECDGVGILGIAWAFGGMIFILVYCTAGISGGHINPAV 111(180)

GmPIP2.1 LYVTVATVIGHKKQT-------GPCDGVGLLGIAWAFGGMIFVLVYCTAGISGGHINPAV 98(180)

GmPIP2.2 LYVTVATIIGHKKQT-------GPCDGVGLLGIAWSFGGMIFVLVYCTAGISGGHINPAV 101(180)

GmPIP2.7 LYVTILTVIGYNHQTATGS--PDLCNGVGVLGIAWAFGGMIFVLVYCTAGISGGHINPAV 109(180)

GmPIP2.8 LYVTILTVIGYNHQTAT-A--AEPCSGVGVLGIAWAFGGMIFVLVYCTAGISGGHINPAV 108(180)

GmPIP1.1 LYITVLTVMGVFKS-------KSKCSTVGIQGIAWAFGGMIFALVYSTAGISGGHINPAV 116(180)

GmPIP1.2 LYVTVLTVMGVAKS-------PSKCSTVGVQGIAWSFGGMIFALVYCTAGISGGHINPAV 136(180)

GmPIP1.3 LYITVLTVMGVNRA-------PNKCSSVGIQGIAWAFGGMIFALVYCTAGISGGHINPAV 116(180)

GmPIP1.4 LYITVLTVMGVNRA-------PNKCSSVGIQGIAWAFGGMIFALVDCTAGISGGHINPAV 116(180)

GmPIP1.5 LYITILTVMGVNRS-------PSKCASVGIQGIAWAFGGMIFALVYCTAGISGGHINPAV 117(180)

GmPIP1.6 LYITILTVMGVNRS-------PSKCASVGIQGIAWAFGGMIFALVYCTAGISGGHINPAV 119(180)

GmPIP1.7 LYITVLTVMGVAGA-------KSKCSTVGIQGIAWAFGGMIFALVYCTAGISGGHINPAV 120(180)

GmPIP1.8 LYITILTVMGVNRS-------SSKCATVGIQGIAWAFGGMIFALVYCTAGISGGHINPAV 120(180)

GmNIP1.1 IFAGSASVVVN----------KNNNNVVTLPGISIVWGLVVMVLVYSVGHISGAHFNPAV 100(180)

GmNIP1.2 IFAGCASVVVN----------KNNNNVVTHPGISIVWGLVVMVLVYSVGHISGAHFNPAV 100(180)

GmNIP1.3 IFAGCASVVVN----------LDKDKVVTQPGISIVWGLTVMVLVYSVGHISGAHFNPAV 103(180)

GmNIP1.4 IFAGCASVVVN----------LDKDKVVTQPGISIVWGLTVMVLVYSVGHISGAHFNPAV 97(180)

GmNIP1.5Nod26 IFAGCASLVVN----------ENYYNMITFPGIAIVWGLVLTVLVYTVGHISGGHFNPAV 100(180)

GmNIP4.1 IFAGCCSVIINNA--------EETKGRITFPGICLVWGFSVTILVYSLAHVSGAHFNPAV 89(180)

GmNIP6.1 MFAAIGTAIVN----------QKTHGSETLIGCAAANGLAVMIIIFSTGHISGAHLNPAV 139(180)

GmNIP6.2 MFAGTAAAIVN----------QKTNGSETLIGCAATTGLAVMIVILATGHISGAHLNPAV 138(180)

GmNIP5.1 IFAATAGPIVN----------NKYNGVESLMGNAACAGLTVMFIILSIGHISGAHLNPSL 104(180)

: : : : :**.*.**::

**TM3**

**HB**

AtTIP1.1 TFGAFIGGNITLLR------GILYWIAQLLGSVVACLILKFATGGLAVPAFG-----LSA 137(240)

GmTIP1.4 TFGAFIGGHISLLR------GILYWIAQLLGSVVACLLLKFATGGLETSAFS-----LSP 137(240)

GmTIP1.6 TFGAFVGGHITLFR------SILYWIAQLLGSVVACLLLKFATGGLETSAFA-----LSP 137(240)

GmTIP1.1 TFGAFIGGNITLLR------SILYWIAQLLGSVVACILLKSATGGMETTGFS-----LSP 137(240)

GmTIP1.2 TFGAFIGGNITLLR------SILYWIAQLFGSVVACILLKHATGGMETSGFS-----LSP 137(240)

GmTIP1.3 TFGAFIGGNITLLR------SILYWIAQLLGSVVACILLKVATGGMETSAFS-----LSS 137(240)

GmTIP1.9 TFGAFVGGNLTLLR------CVLFWIAQILGSVIACLLLKFITGGQDVPVFK-----LSS 134(240)

GmTIP2.1 TFGLAIGGNITLIT------GFLYWIAQLLGSIVACLLLNLIT-AKSIPSHS-----PAN 134(240)

GmTIP2.2 TFGLAIGGNITLIT------GFLYWIAQLLGSIVACLLLNFIT-AKSIPSHA-----PAT 134(240)

GmTIP2.3 TFGLALGGHITILT------GFFYWIAQLLGSIVACFLLNYVTGGLPTPIHS-----VAS 135(240)

GmTIP2.4 TFGLALGGHITILT------GFFYWIAQLLGSIVACFLLNYVTGGLPTPIHS-----VAS 135(240)

GmTIP2.5 TFGLALGGHITILT------GLFYWIAQLLGSIVASLLLKFVTG-YDTPIHS-----VAA 134(240)

GmTIP2.6 TFGLALGGHITILT------GLFYWIAQLLGSIVASLLLKFVTG-YDTPIHS-----VAA 134(240)

GmTIP2.7 TFGLALGGHITILT------GLFYWIAQLLGSIVASLLLKFVTG-YDTPIHS-----VAA 134(240)

GmTIP5.1 TFAMAVGGHISVPT------ALFYWVAQLIASVMACLVLRVIVVGMHVPTYT-----IAE 138(240)

GmPIP2.10 TFGLFLARKVSLIR------AIMYMVAQCLGAICGVGLVKAFQK-AYYNRYGGGANELSE 161(240)

GmPIP2.11 TFGLFLARKVSLIR------AIMYMVAQCLGAICGVGLVKAFQK-AYYNRYGGGANELSE 161(240)

GmPIP2.9 TFGLFLARKVSLIR------AIMYMVAQCLGAICGVGLVKAFQK-AYYNRYGGGANELSE 161(240)

GmPIP2.12 TFGLFLARKVSLIR------AIMYMVAQCLGAMCGVGLVKAFQK-AYYNRYGGGANELSE 161(240)

GmPIP2.13 TFGLFLARKVSLIR------AIMYMVAQCLGAICGVGLVKAFQK-SYFNKYGGGANSLAA 172(240)

GmPIP2.14 TFGLFLARKVSLIR------AIMYMVAQCLGAICGVGLVKAFQK-SYFNKYGGGANSLAD 158(240)

GmPIP2.3 TFGLFLGRKVSLIR------ALLYMVAQCAGAICGTGLAKGFQK-SYYNRYGGGANSVAD 164(240)

GmPIP2.4 TFGLFLGRKVSLIR------ALLYMVAQCAGAICGTGLAKGFQK-AYYNRYGGGANSVAD 164(240)

GmPIP2.5 TFGLFLGRKVSLVR------ALLYMIAQCAGAICGAGLAKGFQK-SYYNRYGGGVNTVSD 164(240)

GmPIP2.6 TFGLFLGRKVSLVR------ALLYMIAQCAGAICGAGLAKGFQK-SFYNRYGGGVNTVSD 164(240)

GmPIP2.1 TFGLFLARKVSLIR------ALFYMVAQCLGAICGVGLVKAFMK-HSYNSLGGGANSVSA 149(240)

GmPIP2.2 TFGLFLARKVSLIR------AVFYMVAQCLGAICGVGLVKAFMK-HSYNSLGGGANSVSA 154(240)

GmPIP2.7 TFGLFLARKVSLIR------AVGYMVAQVLGAISGVGLVKALQK-SYYNRYNGGVNMLAD 162(240)

GmPIP2.8 TFGLFLARKVSLTR------AVGYMVAQVLGAISGVGLVKALQK-SYYNRYKGGVNMLAD 161(240)

GmPIP1.1 TFGLFLARKLSLTR------AIFYIIMQCLGAICGAGVVKGFE-PHLYERLGGGANTIAK 169(240)

GmPIP1.2 TFGLFLARKLSLTR------TVFYMIMQCLGAICGAAVVKGFQ-SNQYERLGGGANTLSK 189(240)

GmPIP1.3 TFGLFLARKLSLTR------AVFYIVMQCLGAICGAGVVKGFEGNARYELFKGGANFVSH 170(240)

GmPIP1.4 TFGLFLARKLSLTR------ALFYIVMQCLGAICGAGVVKGFEGNARYELFKGGANFVSH 170(240)

GmPIP1.5 TFGLFLARKLSLTR------ALFYIIMQCLGAICGAGVVKGFEGNARYEMFKGGANFVNS 171(240)

GmPIP1.6 TFGLFLARKLSLTR------ALFYIIMQCLGAICGAGVVKGFEGNANYELFKGGANFVNS 173(240)

GmPIP1.7 TFGLFLARKLSLPR------AIFYIVMQCLGAICGAGVVKGFEGKTKYGALNGGANFVAP 172(240)

GmPIP1.8 TFGLFLARKLSLTR------ALFYMVMQVLGAIVGAGVVKGFEGKTFYGQHNGGANFVAP 172(240)

GmNIP1.1 TIAFASTKRFPL------KQVPVYVVAQVVGSTLASGTLRLLFSGKEAQFSG-----TLP 149(240)

GmNIP1.2 TIAFASTRRFPL------KQVPVYVVAQVVGSTLASATLRLLFSGKETQFSG-----TLP 149(240)

GmNIP1.3 TIAHATTKRFPL------KQVPAYVIAQVVGATLASGTLRLIFNGKNDHFAG-----TLP 152(240)

GmNIP1.4 TIAHATTKRFPL------KQVPAYVIAQVVGATLASGTLRLIFNGKSDHFTG-----TLP 146(240)

GmNIP1.5Nod26 TIAFASTRRFPL------IQVPAYVVAQLLGSILASGTLRLLFMGNHDQFSG-----TVP 149(240)

GmNIP4.1 TLSFAIYRHFPLRLAYIKSTVPLYFIAQVLGSFLASGTLYLLFEVNEKTYFG-----TIP 144(240)

GmNIP6.1 TISFAALKHFPW------KNVPVYIGTQVLASVSAAFALKVVFHP-FMSGGV-----TVP 193(240)

GmNIP6.2 TISFAALKHFPW------KHVPMYIGAQVLASICAGFALKGVYHP-FMSGGV-----TVP 192(240)

GmNIP5.1 TIAFAAFRHFPW------THVPAYIAAQVSASICACYALKGVYHP-FLSGGV-----TVP 152(240)

*:. ... : * .: .

**TM4**

**TM5**

AtTIP1.1 GVGVLNAFVFEIVMTFGLVYTVYATAIDPK---NGSLGTIAPIAIGFIVGANILAGGAFS 194(300)

GmTIP1.4 GVGAANALVFEIVMTFGLVYTVYATAVDPK---KGNLGIIAPIAIGFIVGANILAGGAFD 194(300)

GmTIP1.6 GVEAGNALVFEIVMTFGLVYTVYATAVDPK---KGDLGIIAPIAIGFIVGANILAGGAFD 194(300)

GmTIP1.1 GVSVWNALVFEIVMTFGLVYTVYATAVDPK---KGNVGVVAPIAIGFIVGANILVGGAFD 194(300)

GmTIP1.2 GVSVWNALVFEIVMTFGLVYTVYATAVDPK---KGNAGVVAPIAIGFIVGANILVGGAFD 194(300)

GmTIP1.3 GVSVWNALVFEIVMTFGLVHTVYATTVDPK---KGNVGVIGPIAIGSIVGANILVGGAFD 194(300)

GmTIP1.9 GVGVGNAVVLEMVMTFGLVYTVYATTVDPRSR-RGSLGVMAPIVIGFIVGANVLVGGPFD 193(300)

GmTIP2.1 GVNDLQAVVFEIVITFGLVYTVYATAVDPK---KGSLGIIAPIAIGFVVGANILAAGPFS 191(300)

GmTIP2.2 GVNDFQAVVFEIVITFGLVYTVYATAADPK---KGSLGIIAPIAIGFVVGANILAAGPFS 191(300)

GmTIP2.3 GVGAVEGVVTEIIITFGLVYTVYATAADPK---KGSLGTIAPIAIGFIVGANILAAGPFS 192(300)

GmTIP2.4 GVGAVEGVVTEIIITFGLVYTVYATAADPK---KGSLGIIAPIAIGFIVGANILAAGPFS 192(300)

GmTIP2.5 GIGAGEGVVTEIIITFGLVYTVYATAADPK---KGSLGTIAPIAIGFIVGANILAAGPFS 191(300)

GmTIP2.6 GVGAGEGVVTEIIITFGLVYTVYATAADPK---KGSLGTIAPIAIGFIVGANILAAGPFS 191(300)

GmTIP2.7 GIGAGEGVVTEIIITFGLVYTVYATTADPK---KGSLGTIAPIAIGFIVGANILAAGPFS 191(300)

GmTIP5.1 EMTGFGASVLEGTLTFVLVYTVYAARDPRR----GPMSSTGILVVGLIAGASVLASGPFS 194(300)

GmPIP2.10 GYSTGVGLGAEIIGTFVLVYTVFSATDPKRNARDSHVPVLAPLPIGFAVFMVHLATIPVT 221(300)

GmPIP2.11 GYSTGVGLGAEIIGTFVLVYTVFSATDPKRNARDSHVPVLAPLPIGFAVFMVHLATIPVT 221(300)

GmPIP2.9 GYSTGVGLGAEIIGTFVLVYTVFSATDPKRNARDSHVPVLAPLPIGFAVFMVHLATIPVT 221(300)

GmPIP2.12 GYSTGVGLGAEIIGTFVLVYTVFSATDPKRNARDSHVPVLAPLPIGFAVFMVHLATIPVT 221(300)

GmPIP2.13 GYSTGTGLGAEIIGTFVLVYTVFSATDPKRNARDSHVPVLAPLPIGFAVFMVHLATIPVT 232(300)

GmPIP2.14 GYSTGTGLGAEIIGTFVLVYTVFSATDPKRNARDSHVPVLAPLPIGFAVFMVHLATIPVT 218(300)

GmPIP2.3 GYNNGTALGAEIIGTFVLVYTVFSATDPKRNARDSHVPVLAPLPIGFAVFMVHLATIPIT 224(300)

GmPIP2.4 GYNNGTALGAEIIGTFVLVYTVFSATDPKRNARDSHVPVLAPLPIGFAVFMVHLATIPIT 224(300)

GmPIP2.5 GYNKGTALGAEIIGTFVLVYTVFSATDPKRSARDSHVPVLAPLPIGFAVFMVHLATIPVT 224(300)

GmPIP2.6 GYNKGTALGAEIIGTFVLVYTVFSATDPKRNARDSHVPVLAPLPIGFAVFMVHLATIPVT 224(300)

GmPIP2.1 GYNKGSALGAEIIGTFVLVYTVFSATDPKRSARDSHIPVLAPLPIGFAVFMVHLATIPIT 209(300)

GmPIP2.2 GYNKGSALGAEIIGTFVLVYTVFSATDPKRSARDSHVPVLAPLPIGFAVFMVHLATIPIT 214(300)

GmPIP2.7 GYSKGTGLGAEIIGTFILVYTVFSATDPKRVARDSHVPVLAPLPIGFAVFIVHLATIPIT 222(300)

GmPIP2.8 GYSKGTGLGAEIIGTFILVYTVFSATDPKRVARDSHVPVLAPLPIGFAVFMVHLATIPIT 221(300)

GmPIP1.1 GYTNSAGLGAEIVGTFVLVYTVFSATDAKRNARDSHVPILAPLPIGFAVFLVHLATIPVT 229(300)

GmPIP1.2 GYSKGDGLGAEIVGTFILVYTVFSATDAKRNARDSHVPILAPLPIGFAVFLVHLATIPIT 249(300)

GmPIP1.3 GYTKGDGLGAEIVGTFILVYTVFSATDAKRNARDSHVPILAPLPIGFAVFLVHLATIPIT 230(300)

GmPIP1.4 GYTKGDGLGAEIVGTFILVYTVFSATDAKRNARDSHVPILAPLPIGFAVFLVHLATIPIT 230(300)

GmPIP1.5 GYTKGDGLGAEIVGTFVLVYTVFSATDAKRNARDSHVPILAPLPIGFAVFLVHLATIPIT 231(300)

GmPIP1.6 GYTKGDGLGAEIVGTFVLVYTVFSATDAKRNARDSHVPILAPLPIGFAVFLVHLATIPIT 233(300)

GmPIP1.7 GYTKGDGLGAEIVGTFILVYTVFSATDAKRSARDSHVPILAPLPIGFAVFLVHLATIPIT 232(300)

GmPIP1.8 GYTKGDGLGAEIVGTFILVYTVFSATDAKRSARDSHVPILAPLPIGFAVFLVHLATIPIT 232(300)

GmNIP1.1 SGSNLQAFVIEFLITFFLMFVVSGVATDNR-----AIGELAGIAVGSTVLLNVMFAGPIT 204(300)

GmNIP1.2 SGSNLQAFVIEFLITFFLMFVISGVATDDR-----AIGELAGIAVGSTVLLNVMFAGPIT 204(300)

GmNIP1.3 SGSDLQSFVVEFIITFYLMFVISGVATDNR-----AIGELAGLAVGSTVLLNVMFAGPIT 207(300)

GmNIP1.4 GGSDLQSFVVEFIITFYLMFVISGVATDNR-----AIGELAGLAVGSTVLLNVMFAGPIT 201(300)

GmNIP1.5Nod26 NGTNLQAFVFEFIMTFFLMFVICGVATDNR-----AVGELAGIAIGSTLLLNVIIGGPVT 204(300)

GmNIP4.1 SGSYIQSLVFEILTSFLLMFVVCAVSTDNR-----AIGKLGGIAVGMTIIVNVFIAGPIS 199(300)

GmNIP6.1 SVGYGQAFATEFIVSFILMFVVTAVATDTR-----AVGELAGIAVGATVMLNILIAGPTT 248(300)

GmNIP6.2 SGGYGQSFALEFIIGFNLMFVVTAVATDTR-----AVGELAGIAVGATVMLNILIAGPVS 247(300)

GmNIP5.1 TVSVAQAFATEFIITFILLFVVTAVATDTR-----AVGELAGIAVGATVLLNILISGPTS 207(300)

. * * *:..: .. : . : :* : .

**TM6**

**HE**

AtTIP1.1 GASMNPAVAFGPAVVS---WTWTNHWVYWAGPLVGGGIAGLIYEVFFIN-TTHEQLPTTD 250(360)

GmTIP1.4 GASMNPAVSFGPAVVS---GTWANHWVYWVGPLIGSAIAAIIYETFFITPNSYEHLPVTD 251(360)

GmTIP1.6 GASMNPAVSFGPAVVS---WTWSNHWVYWVGPFAGAAIAAVVYEIFFISPNTHEQLPVTD 251(360)

GmTIP1.1 GASMNPAVSFGPAVVT---WSWTHHWVYWVGPFIGAAIAAVIYDNIFIGDDGHEPLSSSD 251(360)

GmTIP1.2 GASMNPAVSFGPAVVT---WSWTHHWVYWVGPFIGAAIAAIIYDNIFIGDDGHEPLSSSD 251(360)

GmTIP1.3 GASMNPAVCFGPALIN---WSWTHHWVYWLGPFIGSATAAILYDNIFIGDDGHEPLSNSD 251(360)

GmTIP1.9 GASMNPAASFGPAVVG---WSWKNHWVYWVGPLVGGGLAGFMYELIFVSHSRQRFRRSYY 250(360)

GmTIP2.1 GGSMNPARSFGPAVVS---GDLAANWIYWVGPLIGGGLAGLIYGDVFIGSYAPVPASETY 248(360)

GmTIP2.2 GGSMNPARSFGPAVVS---GDFAANWIYWVGPLIGGGLAGLIYGDVFIGSYAAVPASETY 248(360)

GmTIP2.3 GGSMNPARSFGPAVVS---GDFHDNWIYWVGPLIGGGLAGLIYGNVFIRSDHAPLSSEF- 248(360)

GmTIP2.4 GGSMNPARSFGPAVVS---GDFHDNWIYWVGPLIGGGLAGLIYGNVFIRSDHAPLSSEF- 248(360)

GmTIP2.5 GGSMNPARSFGPAVVS---GDFHDNWIYWVGPLIGGGLAGLIYTYAFIPTNHAPLATEF- 247(360)

GmTIP2.6 GGSMNPARSFGPAVVS---GDFHDNWIYWVGPLIGGGLAGLIYTYAFIPTQHAPLATDF- 247(360)

GmTIP2.7 GGSMNPARSFGPAVVS---GDFHDNWIYWVGTLIGGGLAGLIYTYAFIM----------- 237(360)

GmTIP5.1 GGSMNPACAFGSAAIA---GSFRNQAVYWVGPLIGATIAGLLYDNELGSKLCSVMQ---- 247(360)

GmPIP2.10 GTGINPARSLGAAVMYNQQKAWDDHWIFWVGPFIGAAIAAFYHQFILRAGAAKALGSFRS 281(360)

GmPIP2.11 GTGINPARSLGAAVMYNQQKAWDDHWIFWVGPFIGAAIAAFYHQFILRASAAKALGSFRS 281(360)

GmPIP2.9 GTGINPARSLGAAVMYNQQKAWDDHWIFWVGPFIGAAIAAFYHQFILRAGAAKALGSFRS 281(360)

GmPIP2.12 GTGINPARSFGAAVMYNQKKAWDDQWIFWVGPFIGAAIAAFYHQFILRASAAKAVGSFRS 281(360)

GmPIP2.13 GTGINPARSLGAAVIYNQDKPWDDHWIFWVGPFIGAAIAAFYHQFILRAGAAKALGSFRS 292(360)

GmPIP2.14 GTGINPARSLGAAVIYNQDKPWDDHWIFWVGPFIGAAIAAFYHQFILRAGAAKALGSFRS 278(360)

GmPIP2.3 GTGINPARSFGAAVIYNKDKIWDDQWIFWVGPIVGAAVAAFYHQYILRAAAIKALGSFRS 284(360)

GmPIP2.4 GTGINPARSFGAAVIYNEDKIWDDQWIFWVGPIVGAAVAAFYHQYILRAAAIKALGSFRS 284(360)

GmPIP2.5 GTGINPARSFGPAVIFNNDKAWDDQWIYWVGPFVGAAVAAFYHQYILRAAAIKALGSFRS 284(360)

GmPIP2.6 GTGINPARSFGPAVIFNNDKAWDDQWIYWVGPFVGAAVAAIYHQYILRGSAIKALGSFRS 284(360)

GmPIP2.1 GTGINPARSFGAAVIYNNGKVWDDHWIFWVGPFVGALAAAAYHQYILRAAAIKALGSFRS 269(360)

GmPIP2.2 GTGINPARSLGAAVIYNNGKVWDEHWIFWVGPLVGALAAAAYHQYILRAGAIKALGSFRS 274(360)

GmPIP2.7 GTGINPARSLGPAVIFNNEKAWDDQWIFWVGPFIGAAIAAFYHQSVLRAQAAKALGSFRS 282(360)

GmPIP2.8 GTGINPARSLGPAVIFNNEKAWDDQWIFWVGPFIGAALAAFYHQSVLRAQAAKALGSFRS 281(360)

GmPIP1.1 GTGINPARSLGAAIIFNKDQAWDDHWIFWVGPFIGAALAALYHQIVIRAIPFSSK----- 284(360)

GmPIP1.2 GTGINPARSLGAALVYNKDQAWDNHWIFWVGPFIGAALAALYHQIVLRAIPFKSK----- 304(360)

GmPIP1.3 GTGINPARSLGAAIIYNRDHAWDDHWIFWVGPFIGAALAAVYHQIVIRAIPFKTRG---- 286(360)

GmPIP1.4 GTGINPARSLGAAIIYNRDHAWDDHWIFWVGPFIGAALAALYHQIVIRAIPFKTRG---- 286(360)

GmPIP1.5 GTGINPARSLGAAIIYNRDHAWDDQWIFWVGPFIGAALAAVYHQIVIRAIPFKTRA---- 287(360)

GmPIP1.6 GTGINPARSLGAAIIYNRDHAWDDQWIFWVGPFIGAALAAVYHQIVIRAIPFKTRA---- 289(360)

GmPIP1.7 GTGINPARSLGAAIIFNKDLGWDEHWIFWVGPFIGAALAALYHQVVIRAIPFKSK----- 287(360)

GmPIP1.8 GTGINPARSLGAAIIFNKDLGWDDHWIFWVGPFVGAALAALYHQVVIRAIPFKSS----- 287(360)

GmNIP1.1 GASMNPARSIGPAIVHK---EYRGIWIYLVSPTLGAVAGAWVYNSIRYTDKPLREITKSA 261(360)

GmNIP1.2 GASMNPARSIGPAILHN---EYRGIWIYIVSPTLGAVAGTWVYNTIRYTDKPLREITKST 261(360)

GmNIP1.3 GASMNPARSLGPAIVHH---EYRGIWIYLVSPTLGAVAGTWAYNFIRYTNKPVREITKSA 264(360)

GmNIP1.4 GASMNPARSLGPAIVHN---EYKGIWIYLVSPTLGAVAGTWAYNFIRYTNKPVREITKSA 258(360)

GmNIP1.5Nod26 GASMNPARSLGPAFVHG---EYEGIWIYLLAPVVGAIAGAWVYNIVRYTDKPLSEITKSA 261(360)

GmNIP4.1 GASMNPARSLGPALVMW---VYNGIWIYVVGPFVGAILGATCYNLIRYTDKPLREIGASS 256(360)

GmNIP6.1 GSSMNPVRTLGPAIAAN---NYKGIWVYLIAPILGTLCGAGAYTVVKLPEEEATKTPSSA 305(360)

GmNIP6.2 GGSMNPVRTLGPAVAAN---NYKAIWVYLVAPILGALAGAGTYTAVKLPEEDDDAKAKTS 304(360)

GmNIP5.1 GGSMNPVRTLGPAVAAG---NYKHIWIYLVAPTLGALAGAGVYTLVKLRDEE--AEPPRQ 262(360)

* .:**. :*.* :: .. * . :

AtTIP1.1 Y------------ 251(420)

GmTIP1.4 Y------------ 252(420)

GmTIP1.6 Y------------ 252(420)

GmTIP1.1 F------------ 252(420)

GmTIP1.2 F------------ 252(420)

GmTIP1.3 F------------ 252(420)

GmTIP1.9 ------------- 250(420)

GmTIP2.1 P------------ 249(420)

GmTIP2.2 P------------ 249(420)

GmTIP2.3 ------------- 248(420)

GmTIP2.4 ------------- 248(420)

GmTIP2.5 ------------- 247(420)

GmTIP2.6 ------------- 247(420)

GmTIP2.7 ------------- 237(420)

GmTIP5.1 ------------- 247(420)

GmPIP2.10 NPAI--------- 285(420)

GmPIP2.11 NPTI--------- 285(420)

GmPIP2.9 NPTI--------- 285(420)

GmPIP2.12 NPTI--------- 285(420)

GmPIP2.13 NPHN--------- 296(420)

GmPIP2.14 NPHN--------- 282(420)

GmPIP2.3 NA----------- 286(420)

GmPIP2.4 NA----------- 286(420)

GmPIP2.5 NT----------- 286(420)

GmPIP2.6 NA----------- 286(420)

GmPIP2.1 NPTN--------- 273(420)

GmPIP2.2 NPTN--------- 278(420)

GmPIP2.7 SSNL--------- 286(420)

GmPIP2.8 SSNL--------- 285(420)

GmPIP1.1 ------------- 284(420)

GmPIP1.2 ------------- 304(420)

GmPIP1.3 ------------- 286(420)

GmPIP1.4 ------------- 286(420)

GmPIP1.5 ------------- 287(420)

GmPIP1.6 ------------- 289(420)

GmPIP1.7 ------------- 287(420)

GmPIP1.8 ------------- 287(420)

GmNIP1.1 SFLKGVASR---- 270(420)

GmNIP1.2 SFLKGVGRSGSSR 274(420)

GmNIP1.3 SFLKGSEAE---- 273(420)

GmNIP1.4 SFLKGGEAE---- 267(420)

GmNIP1.5Nod26 SFLKGRAASK--- 271(420)

GmNIP4.1 KIFKTSACTSAT- 268(420)

GmNIP6.1 PNGSFRR------ 312(420)

GmNIP6.2 ISSFRR------- 310(420)

GmNIP5.1 VRSFRR------- 268(420)

**Figure S5. Alignments of putative amino acids sequences of GmMIPs transporting non-aqua substrates.**

Multiple alignments were performed using ClustalW2 for upto 30 sequences ([http://www.ch.embnet.org/software/ClustalW.html](http://www.ebi.ac.uk/Tools/clustalw2/index.html)) and ClustalW-XXL for >30 sequences (<http://www.ch.embnet.org/software/ClustalW-XXL.html>). Trans-membrane helices (TM1-TM6) and the two short helices forming the two NPAs (HB and HE) (shaded), NPA motifs (shown in red) and specificity determining positions (SDPs; highlighted in yellow) are also indicated. The numbers at the end of each line indicate amino acid position excluding gaps while those in parentheses include gaps.
